# Assessment of water quality in the piped water supply system by using Water Quality index method

**DOI:** 10.12688/f1000research.156276.2

**Published:** 2025-04-22

**Authors:** Kshyana Prava Samal, Ashok Kumar Tarai

**Affiliations:** 1 Associate Professor & Associate Dean Academics,School of Civil Engineering, Kalinga Institute of Industrial Technology, Bhubaneswar, Odisha, 751024, India; 2 Research Scholar, School of Civil Engineering, Kalinga Institute of Industrial Technology, Bhubaneswar, Odisha, 751024, India

**Keywords:** Drinking water, Heavy metal contamination, SDG 6, Supply water, Water quality parameter, Water quality index

## Abstract

**Background:**

Drinking water of the right and approved quality is a basic requirement for the development of any civilization. According to SDG 6 it is crucial to provide every citizen with equitable water quality and quantity.

**Methods:**

The study area is the smart city of Bhubaneswar with 67 wards and three zones: the north zone, south-west zone, and south-east zone with around 12.4 lakhs population. To maintain the water quality in the supply pipeline in this city, which is always assumed to be safe, it needs to be examined within certain time intervals to check the contamination. In this context, studies on water quality parameters in the supply pipeline network from different anticipated vulnerable areas have been collected for testing. In this context, water samples were collected from areas near industries, market complexes, educational institutions, and construction sites of each ward of Bhubaneswar. Nearly 10 water quality parameters were tested and analyzed using the weighted arithmetic water quality index method. This method takes input of all the parameters and provides the overall water quality index value, which classifies the water in different grades like excellent, good, poor, and very poor quality.

**Results:**

According to the WHO (2011) guidelines, the study found that there is a deviation in the parameters like 10.78% in pH, 19.48% in dissolved oxygen, 43.88% in conductivity, and 22.95% in hardness from the standard limit, but the overall water quality index indicates the water is not in the poor and very poor range. Also, water quality index values identify that the water in the south-east zone is excellent compared to the north and south-west zones. The reason for the slight deterioration is due to the underground cable works, road works and also some areas where the old pipeline system is still existing due to personal encroachment of people as per Public health Engineering department.

## 1. Introduction

The global water consumption has expanded, and this value will continue to increase over the next 50 years. To fulfill Goal 6 of the Sustainable Development Goals, which calls for universal access to clean water and sanitation, safe and reasonably priced drinking water must be provided by 2030. According to the Honorable Minister of Urban Development, Bhubaneswar topped the list of the top 20 smart cities out of 98 nominated cities. Bhubaneswar was selected under the Smart City Development Plan of the Indian Government. The primary mission of a smart city is to fulfill citizens’ desired aspirations in a smart and sustainable manner. Thus, it is worthwhile to determine the extent of water quality detrimental across the wards of Bhubaneswar and intervene in a suitable way to ensure that the SDGs are met. It has been observed that cities like Bhubaneswar with large populations depend more on surface water than on groundwater. Water quality can deteriorate during distribution because of several variables, including interactions within the bulk water or between the distributed water and pipe material ( Kirmeyer et al. 2001). Furthermore, the breakdown of disinfectants, such as chlorine, can deteriorate the microbiological conditions of the distribution system, putting customers’ health at risk ( Clark and Haught, 2005).

Due to rapid urbanization, people’s concern for health issues, and the higher aspiration level of consumers in Bhubaneswar, the government has focused more on water supply for human consumption ( Government of Odisha, 2013). Domestic water demand is growing dramatically in Bhubaneswar because of the twin phenomena of population growth and rural-urban migration, which enhance industrialization, market segmentation, construction, etc. Nowadays, owing to population growth, a huge gap is created between population and water sources, leading to the rapid expansion of various water networks. A potable piped water supply with access to good quality at a low cost, equitable, sustainable, and environmentally acceptable is urgently needed. The aging and deterioration of pipeline systems in the distribution has now become a challenge to overcome ( Shannon et al., 2008).

Although the piped water supply has been implemented in Bhubaneswar, a mid-sized city and state capital of Odisha, India, the question arises as to whether equity in quality with real sense has been achieved. Because the public comes from a variety of professional backgrounds, it is important to raise awareness about the quality of the water supply ( Nasirian, 2007). The water quality index (WQI), which was initially developed by Horton in 1965, has been widely used by many researchers. The WQI is an all-inclusive instrument that combines several supply water chemical constituents in a comprehensible format. This is accomplished by using an aggregation function and selectively weighing the water quality factors ( Abtahi et al. 2015). Moreover, the Water Quality Index (WQI) has demonstrated efficacy in disseminating water quality data to decision makers, as evidenced by its extensive implementation in numerous water quality assessment ( Batabyal and Chakraborty, 2015; Chen et al., 2019).

The Water Quality Index (WQI), frequently employed by researchers in developing nations, utilizes the Weighted Arithmetic Water Quality Index Method (WAWQIM). This strategy is especially common in areas with inadequate infrastructure for comprehensive data collection ( Yogendra & Puttaiah 2008). This study employed the Water Quality Index (WQI) as a tool to scrutinize water quality and its variations ( Samal et al., 2023).

Assuming that the piped water supply system is consistently of high quality and safe, several investigations on its quality have been conducted. We measured some of the critical quality characteristics at 335 locations that received piped water supply in order to determine the answer to this issue. The water quality parameters studied include hardness, pH, chloride, dissolved oxygen (DO), alkalinity, electrical conductivity (EC), total dissolved solids (TDS), biochemical oxygen demand (BOD), nitrate, sulfate, lead, copper, and zinc.

Few studies on the quality measurement of supply water have assumed that supply water quality deteriorates with the aging of infrastructure, type of pipe materials, and the combination of wastewater and drinking water ( Liu et al., 2017). Potable water needs to maintain its quality according to the guidelines of the World Health Organization ( WHO, 2011) and BIS ( Bureau of Indian Standards: IS 10500:2012). Sometimes, these factors may lead to a loss in the quality of the piped water supply. It was observed that most of the leak points were detected as damages and cracks owing to the laxity of contractors during installation and pipe fittings. In addition, most of the leak points were detected at the connection points of couplings, elbows, valves, and saddle clamps. Backfilling and bedding sand containing large and sharp stones causes pipes and fittings to scratch and puncture ( Qahtani et al., 2020). Furthermore, it was also found that the water quality deteriorated when synthetic materials were used in water pipes, particularly when trace organics were leached out by the flowing water. After testing, the presence of carbon tetra chloride (CTC), toluene, chloroform, styrene, and O-xylene were also detected in the water pipeline system ( Shaikh et al., 2019). The variation in dissolved oxygen is directly related to the corrosion rate through redox couple reactions in pipeline systems. Dissolved oxygen consumption is related to pipeline materials such as copper, steel, and iron ( Vargas et al., 2010).

Biochemical oxygen demand was used to calculate the total amount of oxygen consumed over an extended period of time by bacteria and other microorganisms involved in stabilizing decomposable organic matter. Biochemical oxygen demand tests are widely used to assess the effects of effluents from sources, such as pulp mills, municipal wastewater treatment facilities, feedlots, and vegetation used in food processing, which can include large amounts of biodegradable organics. According to Dey et al. (2021), an increased oxygen demand indicates the possibility of an increase in dissolved oxygen as the organic content in the sewage is oxidized by macrobiotics. The extremely low oxygen demand indicates the presence of toxic or nonbiodegradable contaminants in pure water. When the biochemical oxygen demand of drinking water ranges from 1 to 2 ppm, it is considered reasonably clean; when it falls between 3 and 5 ppm, it is considered moderately clean. The biochemical oxygen demand of contaminated water is 6–9 ppm. A high level of pollution is indicated by a biochemical oxygen demand of more than 10 ppm in water ( Koda et al., 2017).

Corrosion and water quality degradation in water distribution systems are significantly influenced by the pipe type used. A combination of unlined cast iron pipes and galvanized pipes was used to study the characteristics of the deposited scale and water quality in the water distribution system. Bench testing was conducted to assess water quality parameters, such as pH, dissolved oxygen (DO), oxidation and reduction potential, alkalinity, conductivity, turbidity, color, Fe2+, and Zn ^2+^. It was found that the significant effect of the pipe material on the corrosion scale characteristics leads to water quality variation. Iron, which is primarily released as ferric particulates from pipe lines, affects pH and alkalinity levels ( Li et al. 2016). Learbuch et al. (2021) observed the impact of pipe material on the microbial population and biofilm development in semi-stagnant conditions. It was deduced that pipe material plays an important role in the growth of biomass concentration, the increase of specific microorganisms, and the bacterial community composition in the distribution system with unchlorinated drinking water.

## 2. Methods

### 2.1 Study area

Bhubaneswar is situated at 20 °12 n to 20 °25’ N latitude and 85 °44’E to 85 °55’E longitude across the main axis of eastern Ghats in the Khordha district of Odisha on the western flange, as shown in Figure 1a (also refer underlying data). Bhubaneswar is the capital city of eastern Odisha. The rich heritage associated with the city, the spread of religious spots (Bhubaneswar is referred to as Temple City), contemporary acknowledgement as an education, and IT hub have contributed significantly to the growth and publicity of the city.

**Figure 1a.**
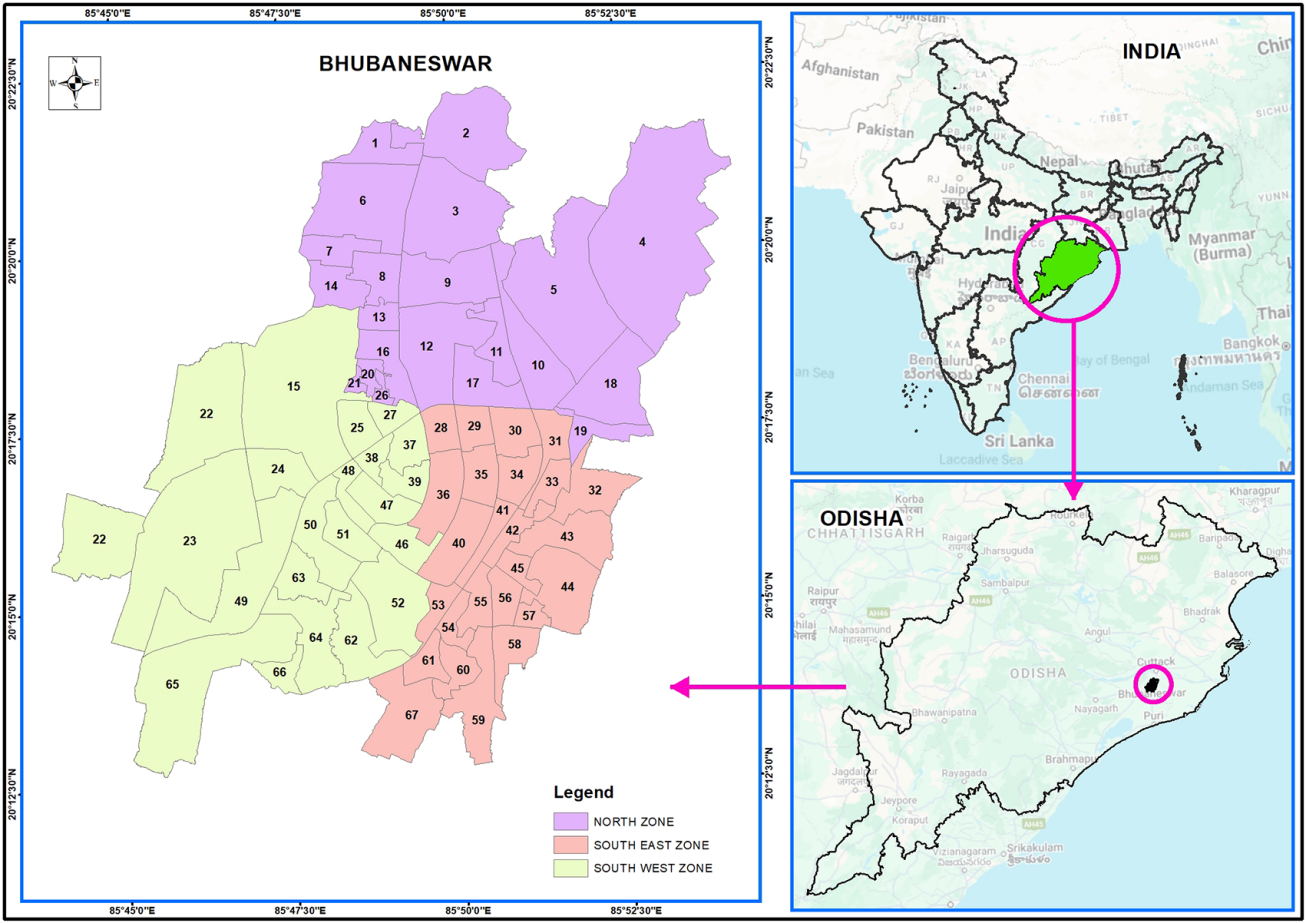
Bhubaneswar index map.

The BMC (Bhubaneswar Municipality Corporation) has separated the city of Bhubaneswar into three zones. There were 21 wards in the north zone, 21 in the south-west zone, and 25 in the south-east zone. The total population in all of these wards was approximately one million. The study concentrated on piped supply water in the northern, South-West, and South-East zones of Bhubaneswar, which comprises 67 wards, as per Bhubaneswar Municipal Corporation ( BMC, 2011). The supply water sample collection areas were emphasized, keeping the following vulnerable areas in mind: industrial areas, market complexes, high-density populations, construction sites, and academic institutions. The water quality parameters for Bhubaneswar were tested zone-wise in the laboratory according to the guidelines stated in the BIS ( Bureau of Indian Standards: IS 10500:2012). Water samples were taken at random from the 67 wards listed above, and the results are shown as ward-wise data. The detail flow chart of the process is mentioned in the Figure 1b. In each ward, five locations were selected, keeping in mind the areas with the presence of more population, new ongoing constructions, more academic institutions or industries, and the presence of market hubs. The areas were selected keeping in mind that there is a greater chance of contamination in comparison to other areas due to the multiusers in one place and the chance of more wear and tear, corrosion, leakage, etc. of the water pipeline system.

**Figure 1b.**
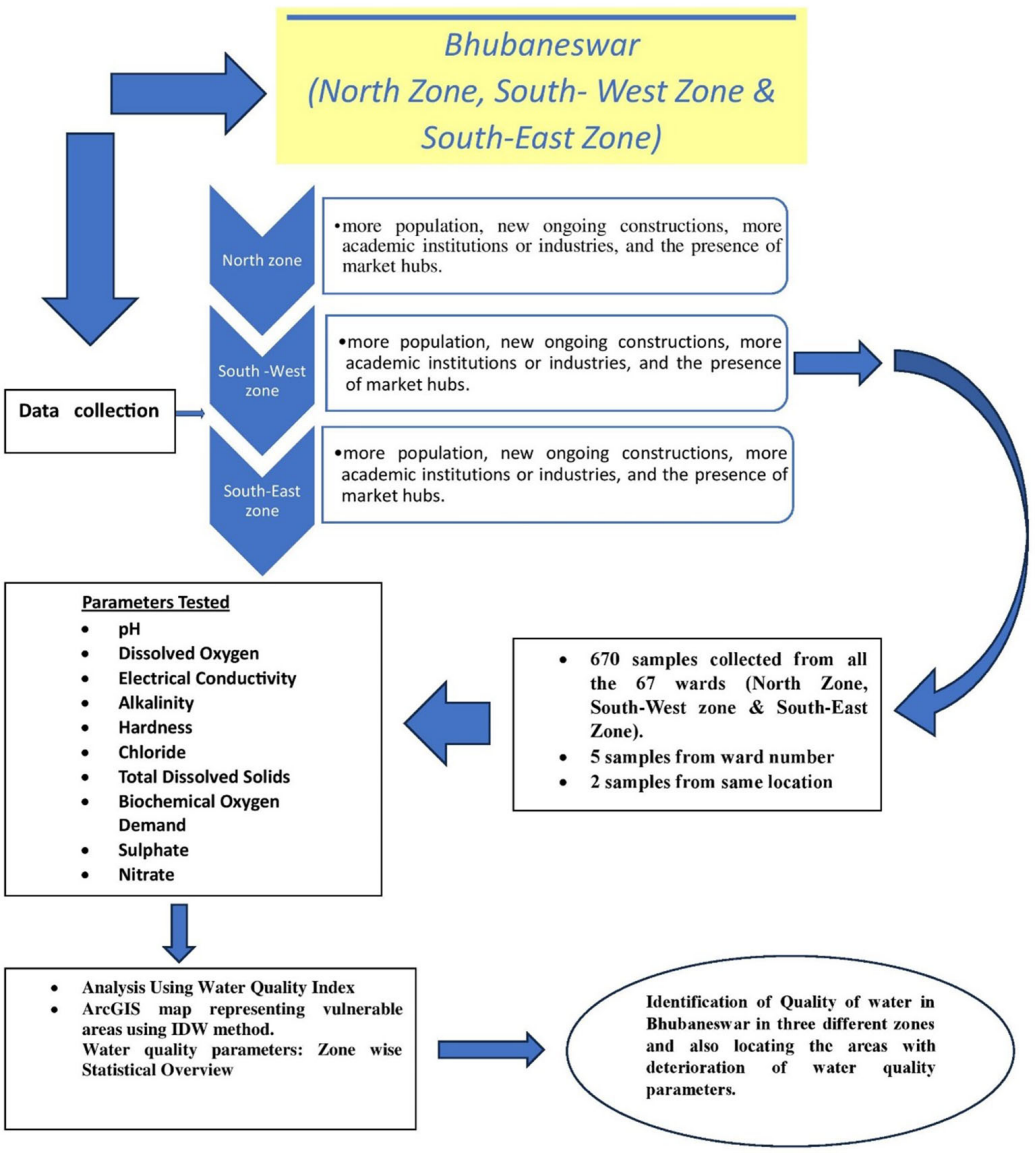
Flow chart of the detail study.

These parameters were selected by reviewing previous studies on the quality of potable water. Supply water samples were collected with a sample size of approximately 335 from different locations and two samples from the same location. The samples were kept at approximately 5 °C in glass bottles with a one liter capacity for analysis. From each location, two water samples were collected in clean screw-tight bottles. The testing procedure followed the American Public Health Association (APHA) book. Parameters such as sulfate, nitrate, lead, copper, and zinc were obtained from the State Pollution Control Board Laboratory in Bhubaneswar. Physicochemical parameters, including pH, dissolved oxygen, conductivity, alkalinity, hardness, chloride, total dissolved solids, biochemical oxygen demand, sulfate, nitrate were tested using the standard methods in APHA (1926). The weighted arithmetic index approach was used to calculate the water quality index by considering the average value of each parameter ( Brown et al., 1970).

The water supply system in Bhubaneswar dates back to 1954, with multiple water treatment plants (WTPs) established over the years. The system has been augmented to meet the growing demand of the city. In 1962, another Treatment Plant was commissioned at Palasuni, Bhubaneswar sourcing required water from River Kuakhai.

**Figure 2.**
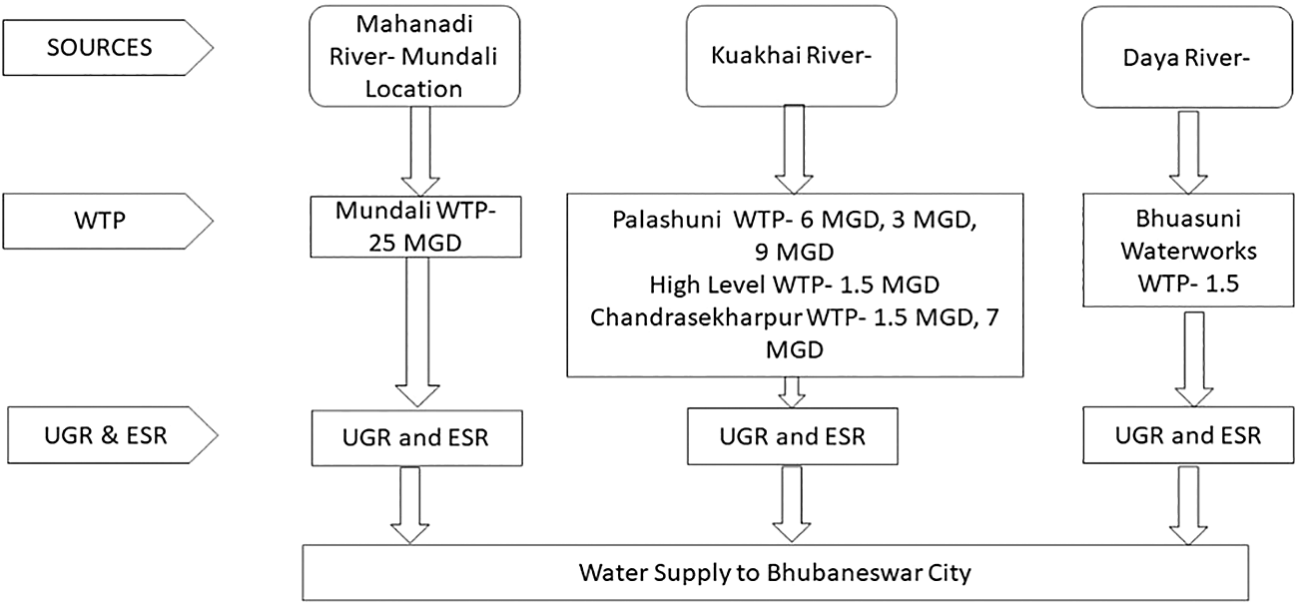
Schematic diagram of Bhubaneswar water supply system.

Bhubaneswar City mostly depends on surface water sources such as the Mahanandi, Kuakhai, and Daya rivers. At present, surface water is being supplied amounting to 24.50 MLD from Daya, 110.47 MLD from Kuakhai Rive, 101.25MLD from River Mahandai (Mundali Intake).Apart from the above, there are number of production wells from which water is being supplied to certain core areas as well as fringe areas to meet the demand.A new WTP of 130MLD is being constructed to augment the treated water capacity of the city, for which water will be drawn from Mahanadi river at Mundali.The intake points are located on the banks of these rivers. Thereafter, the treated water is transmitted to the mass-balancing reservoir, the underground reservoir (UGR), which is pumped to the elevated service reservoir (ESR). House-level connections receive water from elevated service reservoirs through gravity. A schematic of these sources is presented in Figure 2.

Groundwater is being extracted by households to meet the demand of drinking water. There is possibility of geogenic contamination of groundwater which may impact overall human health. The lack of proper regulation on groundwater extraction results in overexploitation in Indian cities (Suhag, 2016). As a result, utilities supplement surface water from distant sources, requiring significant investments in transmission infrastructure development (Larsen et al., 2016). The study conducted by World Resource Institute in the year 2020 classifies cities based on surface water availability index into six categories, (i) extremely low (-0.149 to -0.025); (ii) low (-0.024 to 0.044); (iii) low to medium (0.045 – 0.122); (iv) medium to high (0.123–0.198); high (0.199 – 0.275) and extremely high (0.276 – 0.389). The cities like Bhubaneswar, Kolkata, Indore, Kochi are having surface water availability index between 0.199 to 0.278.

**2.1.1 Use of ArcGIS Software for identification of contaminated areas**

This study applied ArcGIS software to identify the topographical features of possible contaminated areas through spatial analysis and visualization. The advanced functionalities of ArcGIS facilitate a comprehensive understanding of environmental degradation and strengthen environmental management strategies. To explore the spatial variation in water quality parameters over the area of Bhubaneswar, the inverse distance weighted interpolation technique was used to produce different maps using the ArcGIS tool with scaling of the concentration of water quality parameters.

**2.1.2 Interpolating surfaces in ARCGIS by Inverse distance-weighted interpolation (IDW)**

Spatial distribution maps were used to illustrate the variations in water quality parameters across Bhubaneswar city. These maps were generated using ArcGIS 10.2.1 software, which offers a range of spatial interpolation techniques. While no single method is universally accurate for all types of studies (Bronowicka-Mielniczuk et al., 2019), research has shown that Kriging methods, particularly the Ordinary Kriging (OK) method, tend to provide more precise prediction surfaces compared to other approaches (Coulibaly and Becker, 2007; Shamsudduha, 2007; Ohmer et al., 2017). However, the Inverse Distance Weighting (IDW) method is the most commonly used deterministic technique, often compared with Kriging methods (Li and Heap, 2011; Bronowicka-Mielniczuk et al., 2019). The IDW method is particularly suitable for analysing data with low spatial autocorrelation (Jie et al., 2013). As a result, the spatial autocorrelation of the water quality values was taken into account when selecting the most appropriate method, which in this case was IDW.

ArcGIS Spatial Analyst offers several interpolation tools for generating surface grids from point data. IDW method is used, when the set of points are dense enough to capture the extent of local surface variation needed for analysis. This is one of the simplest and most readily available methods. The method assumes that a weighted average of values at points within a certain cut-off distance or from a given number m of the closest points (typically 10 to 30) can approximate the value at an unsampled point. Weights are usually inversely proportional to a power of distance ( Sajil et al., 2022; Watson 2013), which is shown in Equation 1.

$$\lambda_{i}=\frac{{D_{i}}^{-a}}{\sum_{i=1}^{n}{D_{i}}^{-a}}$$ (1)

where *λ*_*i*_ is the weight of the unknown point; *D*_*i*_ is the distance between point *i* and the unknown point; and a is the power of ten of weight. While this basic method is easy to implement and is available in almost any GIS. IDW calculates cell values using a linearly weighted combination of sample points. The assigned weight is a function of the distance of an input point from the output cell location. The greater the distance, the less influence the cell has on the output value.

### 2.2 Analysis of water quality parameters

**2.2.1 Calculation of Water Quality Index (WQI)**

The Water quality Index was determined using drinking water quality criteria recognized by the Bureau of Indian standard (BIS) and the World Health Organization (WHO). According to Olubunmi et al. (2016), the WQI approach is a useful tool for educating the public and policymakers about the quality of the water. It is a unambiguous tool that makes it possible to integrate water metrics that are crucial for the water quality. Here ten parameters like pH, dissolved oxygen, conductivity, alkalinity, hardness, chloride, total dissolved solids, biochemical Oxygen demand, sulphate and nitrate are considered for calculation of water quality index. Parameters like lead copper and zinc are excluded from the calculation because these values are below the range of detectable limit.Given these conditions, the weighted arithmetic index approach ( Brown et al., 1970) is utilized in this work to compute the WQI, which is the most appropriate technique to assess the impact of pollution on supply water.

The Water Quality Index (WQI) ( Inayathulla et al., 2013; Samal et al., 2023) is given as:

$$\mathrm{WQI}=\frac{\sum_{i=1}^{n}q_{i}w_{i}}{\sum_{i=1}^{n}w_{i}},$$

Where,

*q*_*i*_ = quality rating of *i* ^th^ water quality parameter

*w*_*i*_ = unit weight of *i* ^th^ water quality parameter ≃ 1; and *q*_*i*_ relates the value of the parameter in polluted water to the standard permissible value i.e. qi=100(vi−vi0si−vi0)k=1∑i=1n1si

Where, *v*_*i*_ = estimated value of the *i* ^th^ parameter,

*v*_*io*_ = ideal value of the *i* ^th^ parameter

*s*_*i*_ = standard permissible value of the *i* ^th^ parameter as per IS 10500

In most of the cases, *v*_*io*_ = 0 except for pH and dissolved oxygen

For pH, *v*_*io*_ = 7 and for dissolved oxygen, *v*_*io*_ = 14.6 mg/l

The unit weight ( *w*_*i*_), which is inversely proportional to the values of the recommended standards is obtained

as: $w_{i}=\frac{k\;}{\mathrm{si}}$ , Where $k=\frac{1}{\sum_{i=1}^{n}1/\mathrm{si}}$

The rating of the water quality using the above method is shown in Table 1.

**Table 1.** Status of Water Quality Index ( Sinha, Kumar & Singh, 2014).

Water Quality Index Level	Water Quality Status
0-25	Excellent Water Quality
26-50	Good Water Quality
51-75	Poor Water Quality
76-100	Very Poor Water Quality
>100	Unsuitable for Drinking

## 3. Results and Discussion

### 3.1 Water quality parameters: Zone wise Statistical Overview

The statistical summary of water quality parameters and their comparison with the permissible limits indicate that the percentage of samples deviating from the permissible limit as per IS 10500 (2012) are shown in Table 2. The water quality parameters were plotted for the three different zones using box-whisker plots, as illustrated in Figures 6-a, b, c, d, e, f, g, h, i, and j. The corresponding maps with vulnerable and safe areas are shown in Figures 4– 13.

**Table 2.** Summary statistics of water quality parameters and comparison with IS 10500 (2012) standards.

Water quality parameters	Zones	Min.	Max.	Avg.	Standard Deviation	IS 10500 Guidelines	No. of samples exceeded the permissible limit	Percentage of samples exceeded the permissible limit (%)
pH	North	5.56	8.61	7.66	0.70	6.5-8.5	13	12.62
	South-West	5.56	8.61	7.73	0.69		14	13.33
	South-East	6.23	8.59	7.56	0.48		8	6.40
Dissolved Oxygen	North	5.91	8.38	7.50	0.52	6.5-8 mg/l	22	21.35
	South-West	5.91	8.95	7.54	0.54		23	21.90
	South-East	5.91	8.20	7.43	0.49		19	15.20
Electrical Conductivity (EC)	North	58.25	480.30	255.42	90.63	300 μs/cm	33	32.03
	South-West	67.70	519.90	251.01	85.78		29	27.61
	South-East	173.16	581.47	342.97	73.69		90	72
Alkalinity	North	16.00	128.00	58.83	21.85	200 mg/l	-	-
	South-West	16.00	128.00	59.64	21.97		-	-
	South-East	22.00	78.00	46.05	13.75		-	-
Hardness	North	13.12	262.40	118.45	60.24	200 mg/l	14	13.59
	South-West	13.12	229.60	116.96	57.98		11	10.47
	South-East	98.40	268.96	186.09	40.78		56	44.8
Chloride	North	18.46	87.03	33.86	11.65	250 mg/l	-	-
	South-West	18.46	87.03	34.29	10.76		-	-
	South-East	21.30	64.61	38.84	7.61		-	-
Total Dissolved Solids (TDS)	North	37.86	312.20	166.02	58.91	500 mg/l	-	-
	South-West	44.01	337.94	163.16	55.75		-	-
	South-East	112.56	377.96	222.93	47.90		-	-
Biochemical Oxygen Demand (BOD)	North	0.58	4.98	2.54	1.32	<5.0 mg/l	-	-
	South-West	0.58	4.98	2.75	1.26		-	-
	South-East	0.55	4.60	2.39	1.00		-	-
Sulphate	North	3	30	15.57282	8.307334	200 mg/l	-	-
	South-West	3	30	17.14286	8.105588		-	-
	South-East	3	30	16.88	8.048843		-	-
Nitrate	North	0.2	24.9	11.80777	7.346517	45 mg/l	-	-
	South-West	3	30	16.41905	7.595268		-	-
	South-East	3	30	16.88	8.062858		-	-

Table 2 represents the pH levels, with a standard deviation ranging from 0.48 to 0.70 across zones, which demonstrates moderate variability, while the average pH values fall within the recommended range of 6.5 to 8.5. The percentage of samples exceeding the permissible limit was minimum in the southeast zone, that is, 6.40%, and the maximum variation was 13.33% in the southwest zone. Dissolved oxygen levels exhibited moderate variability, with standard deviations ranging from 0.49 0.54. Despite the average values being within the acceptable range of 6.5-8 mg/l. The percentage of samples exceeding the permissible limit was minimum in the southeast zone, that is, 15.20%, and the maximum variation was 21.90% in the south-west zone. Electrical conductivity values, with standard deviations ranging from 73.69 to 90.63, indicate considerable variability in mineral content across zones. While the average electrical conductivity values generally align with the guidelines, the percentage of samples exceeding the permissible limits has a minimum of 27.61% in the southwest zone and a maximum of 72% in the southeast zone. These parameters exhibited varying levels of variability and adherence to guidelines across zones. Hardness and total dissolved solids showed significant standard deviations ranging from 40.78 to 60.24 and from 47.90 to 58.91, respectively, indicating substantial variability in mineral content. The minimum variation in hardness was 44.8% in the east zone and a maximum of 10.47% in the southwest zone. The percentage of samples exceeding permissible limits varied widely across parameters and zones, highlighting the complexity of water quality management.

The average hardness value of each sampled zone has been compared with the standard mentioned by Sawyer and McCarty (1967) and USEPA (1994). It is found that piped water supply is in the range of moderately high hardness. The comparison statement is shown in below Table 3.

**Table 3.** Comparison of hardness parameter.

Sample zone	Field observation (Average value) total hardness as CaCO _3_ (mg/l)	Classification as per (Sawyer and McCarthy 1967)	Classification as per USEPA, 1994
North	118.45	Moderately high	moderately hard
South-West	116.96	Moderately high	moderately hard
South-East	186.09	Hard	very hard

**Figure 3.**
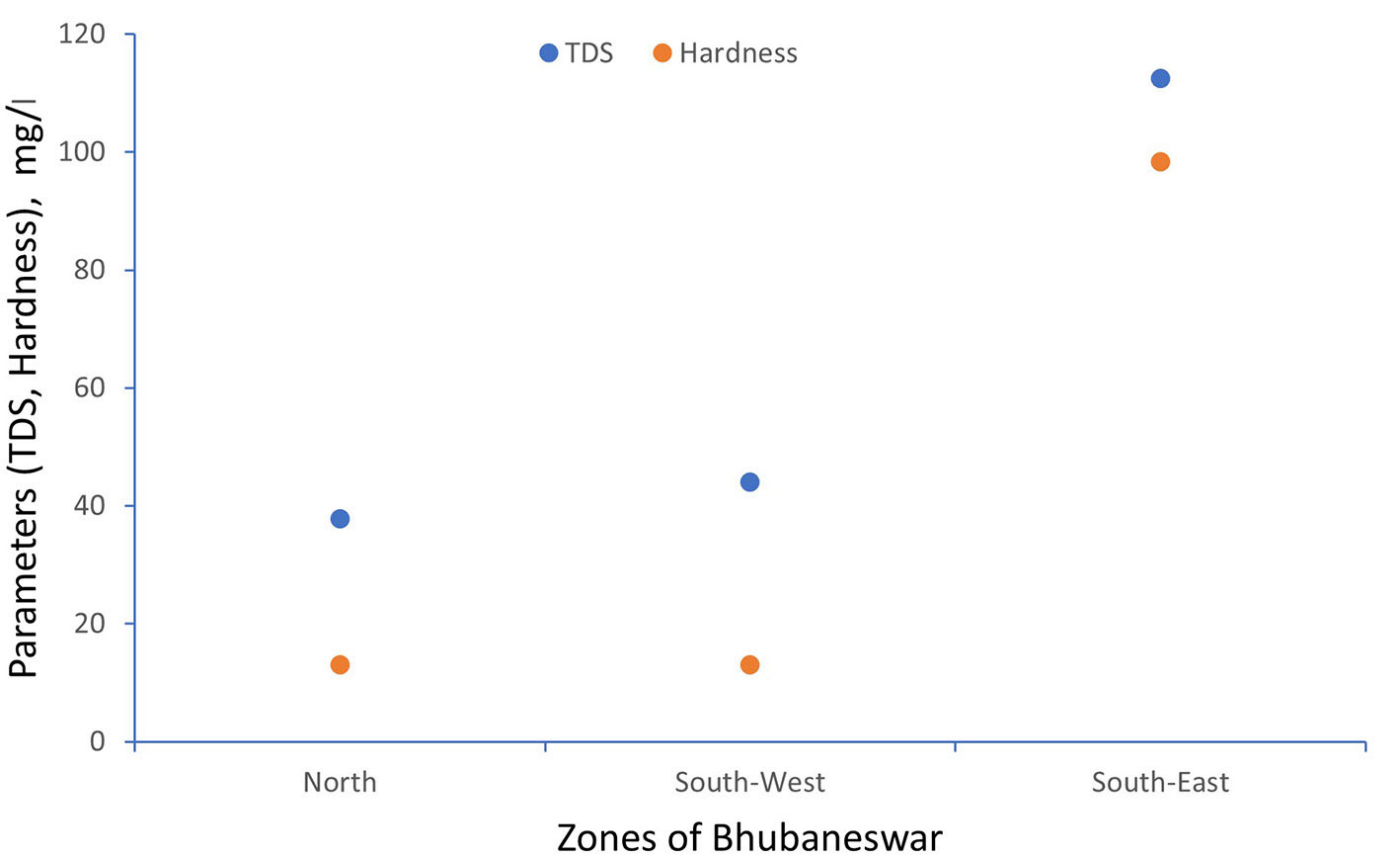
Scatter plot for TDS and Hardness minimum value.

TDS and Electrical conductivity have been tested referring APHA 2022 standard method. The scatter plot between TDS and hardness has been generated for minimum, maximum and average values which are shown in Figure 3 to Figure 5.

**Figure 4.**
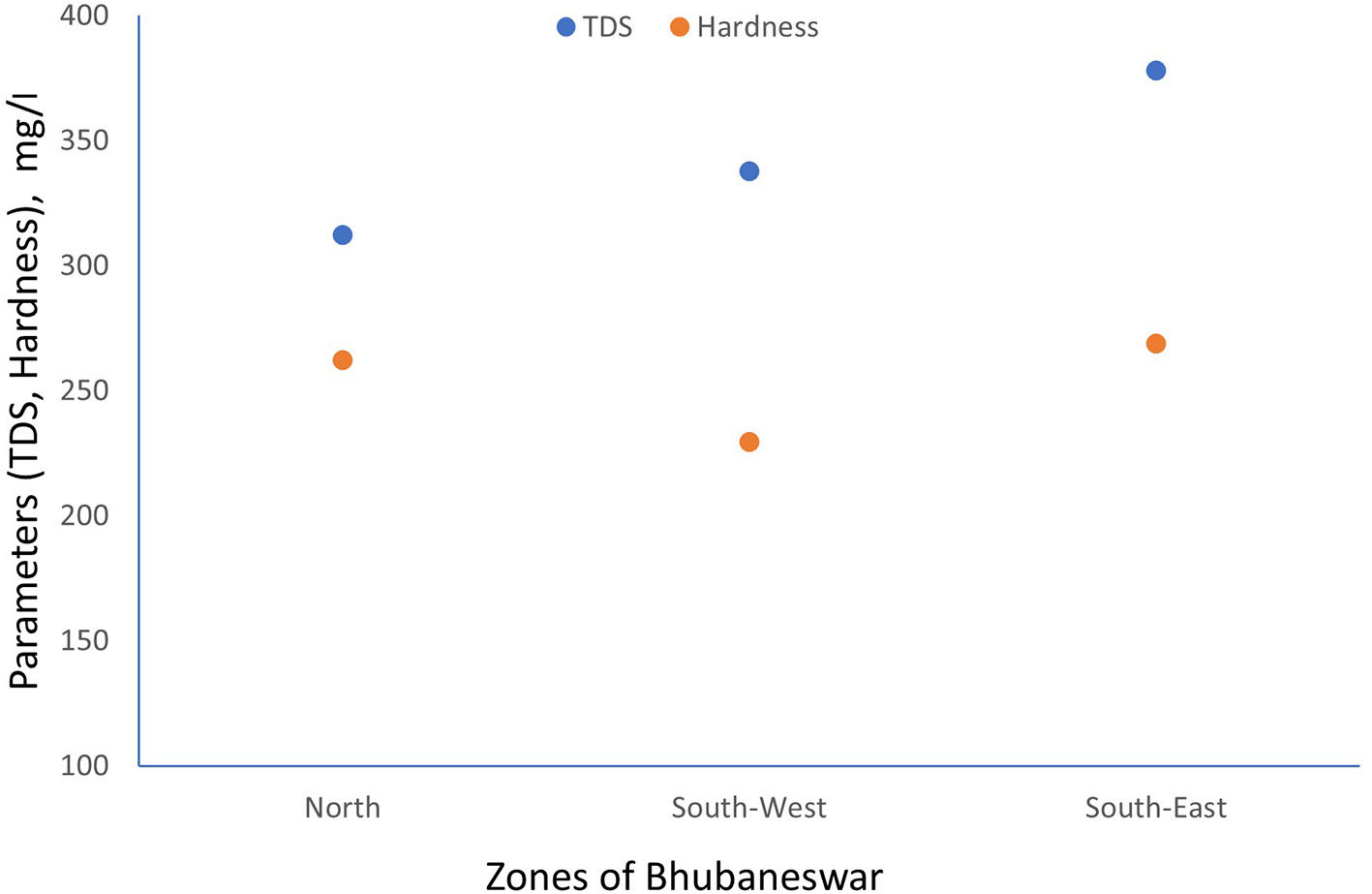
Scatter plot for TDS and Hardness maximum value.

**Figure 5.**
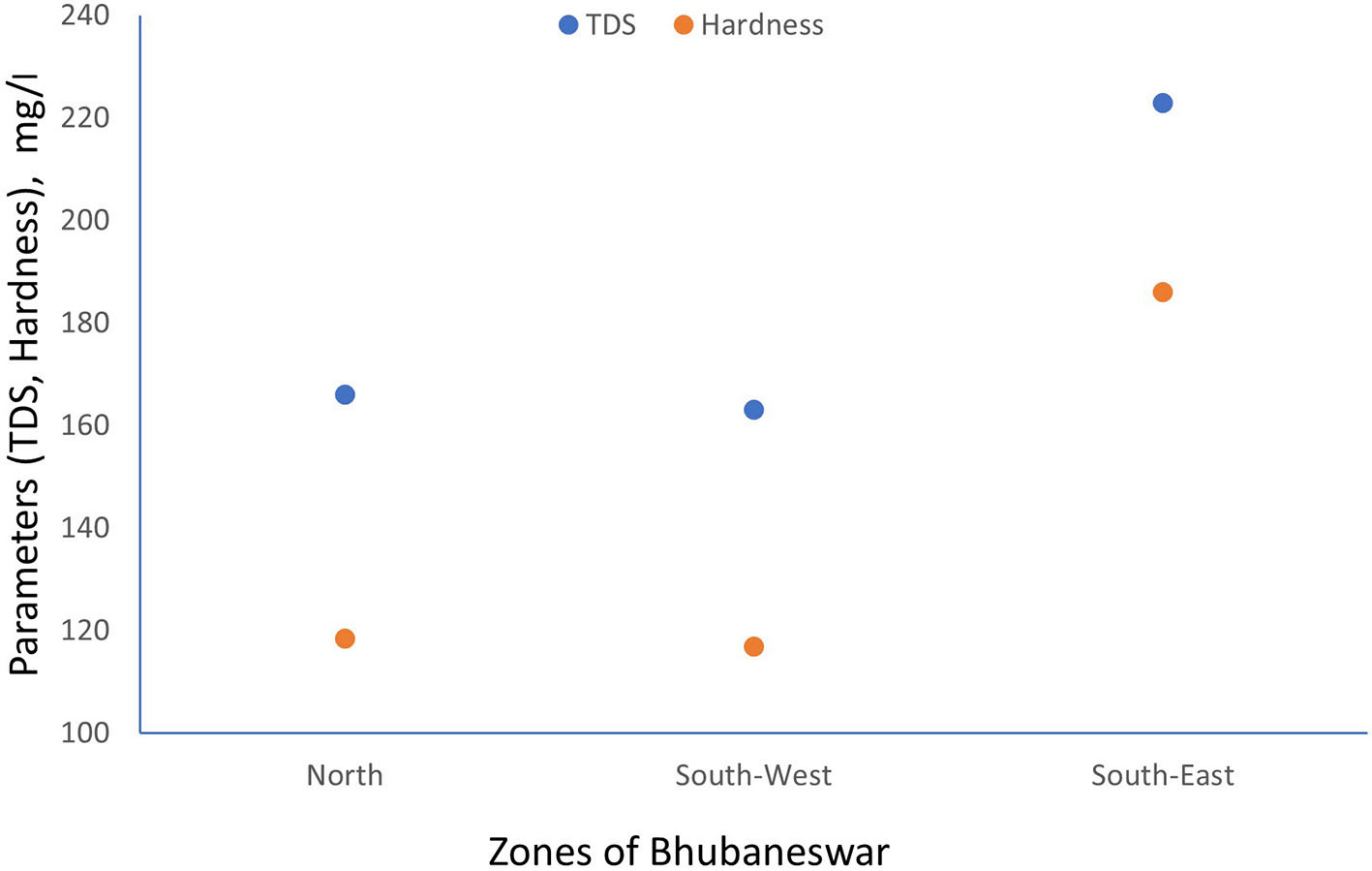
Scatter plot for TDS and Hardness average value.

**Figure 6.**
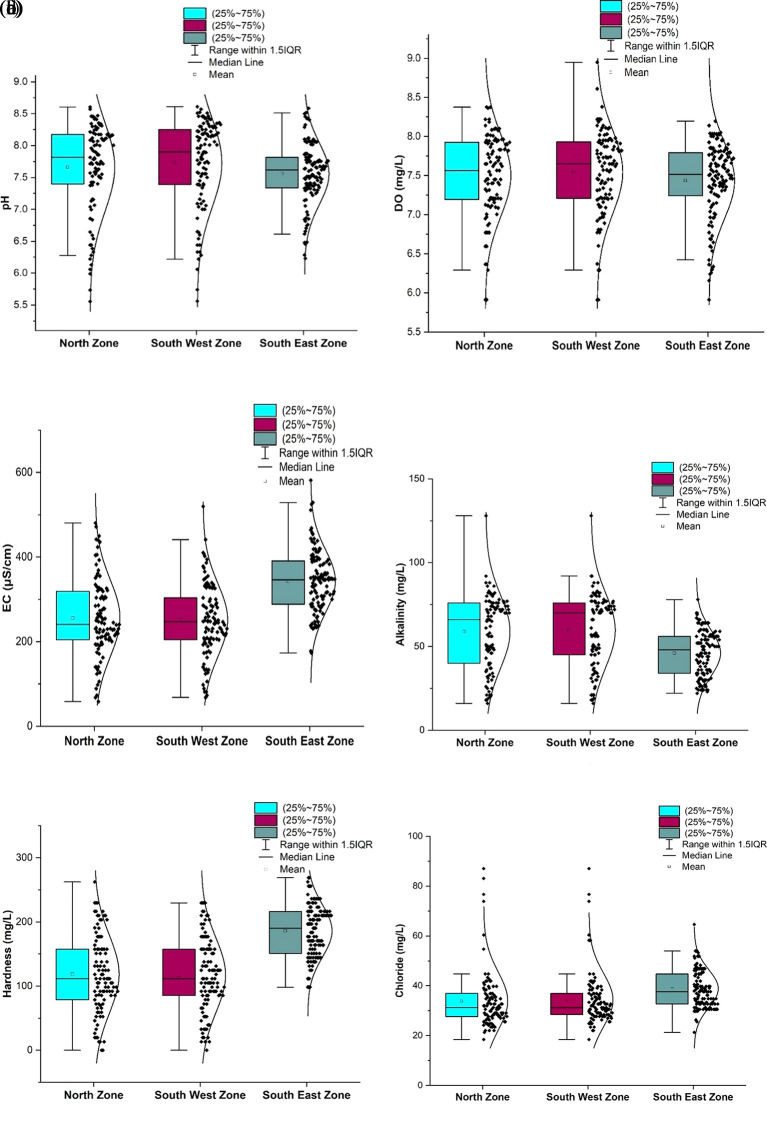

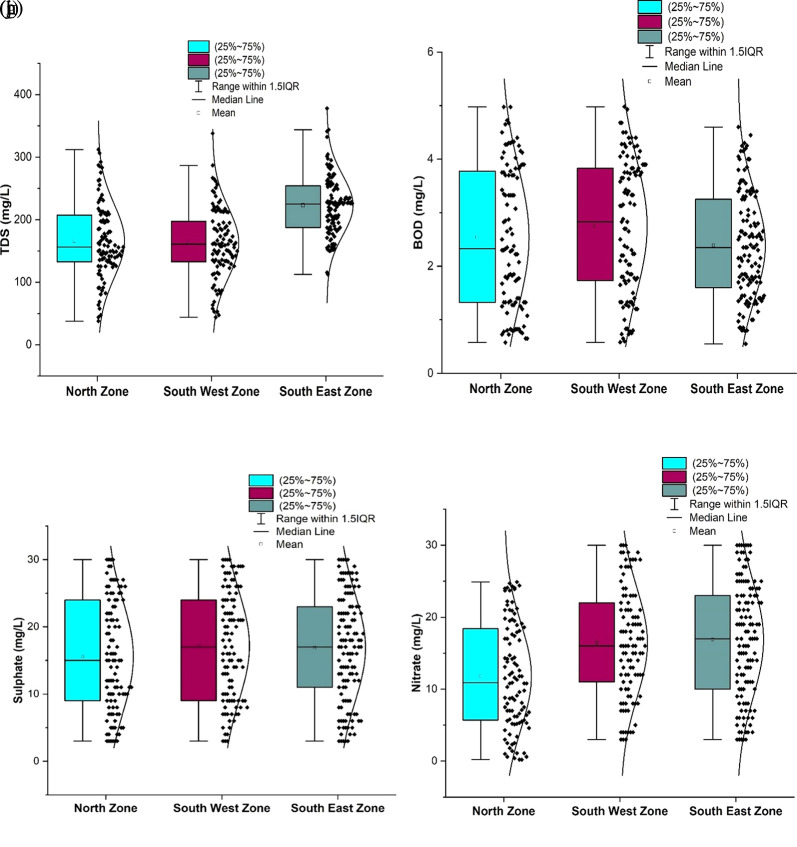
Box-whisker plot represents comparison (a) pH, (b) dissolved Oxygen, (c) electrical conductivity, (d) alkalinity, (e) hardness, (f ) chloride, (g) total dissolved solids, (h) biochemical oxygen demand, (i) sulphate, (j) nitrate between north zone, south-west zone and south-east zone.

Parameters such as alkalinity, hardness, chloride, total dissolved solids, biochemical oxygen demand, sulfate, and nitrate were within permissible ranges. As shown in Figure 6, a comparative analysis was performed for various water quality parameters across three distinct geographic zones: north, south-west, and south-east. Each box-whisker plot represents the distribution and central tendency of a specific parameter within the zones.

### 3.2 Comparative analysis of water quality parameters in North, South-West, and South-East zones

From Table 2 and Figure 6(a) (refer underlying data), it is found that the Bhubaneswar piped supply water has a pH range of 5.56 to 8.61 in both the north and south-west zones, whereas it is 6.23 to 8.59 in the south-east zone. The average values of all three zones were 7.66, 7.73, and 7.56, respectively, indicating that the supply water was slightly alkaline. The contents of hydroxides, carbonates, and bicarbonates primarily regulate the alkalinity of the groundwater ( Sajil Kumar et al., 2013). Piped supply water samples exceeding the permissible limit were 12.62%, 13.33% in the south-west zone, and 6.4% in the south-east zones, respectively. The standard deviation was 0.48 to 0.70, which indicates that the value clustered tightly to the mean. Figure 6(a) represents the areas that are more affected by pH: Damana Area of Ward No. 8 and Indradhanu Market Area of Ward No. 27, whose pH values were 8.61. Areas with acidic water have also been detected, such as Sriram Nagar of Ward No. 59, Swadhin Nagar of Ward No. 34, and Pancha Sakha Nagar of Ward No. 64, with pH values of 5.56, 5.74, 5.74 and 5.56, respectively. The results show that the areas that are affected by this variation in pH are expected to be affected by factors such as the old age of supply pipes (more than 40 years) due to a lack of maintenance and many newly constructed underground sewerage systems, which cause contamination in the pipeline system ( Eun et al., 2011).

Dissolved oxygen values are a significant indicator of water quality and organic pollution in water bodies. Dissolved oxygen had average values of 7.5, 7.54, and 7.43 in the north, south, west, and southeast zones, respectively. There is a deviation of dissolved oxygen values of 21.35%, 21.9%, and 15.2% from the permissible range in the northern, South-west and South-east respectively, as shown in Table 2 and Figure 6(b). In addition, it was found that there were some areas with dissolved oxygen values less than 6 mg/l, such as Sikharchandi Nagar of Word No. 2, Shakti Vihar of Ward No. 20, Shree Vihar of Ward No. 22, Dumduma Phase 3 of Ward No. 64, and Jharapada High School Area of Ward No. 42, as presented in Figure 8 Ward wise dissolved oxygen in supply water of Bhubaneswar, refer underlying data. Typically, polyphosphates are coated inside municipal water supply pipes to shield the metal from oxygen exposure, resulting in high dissolved oxygen concentrations ( Sarin et al., 2004).

**Figure 7.**
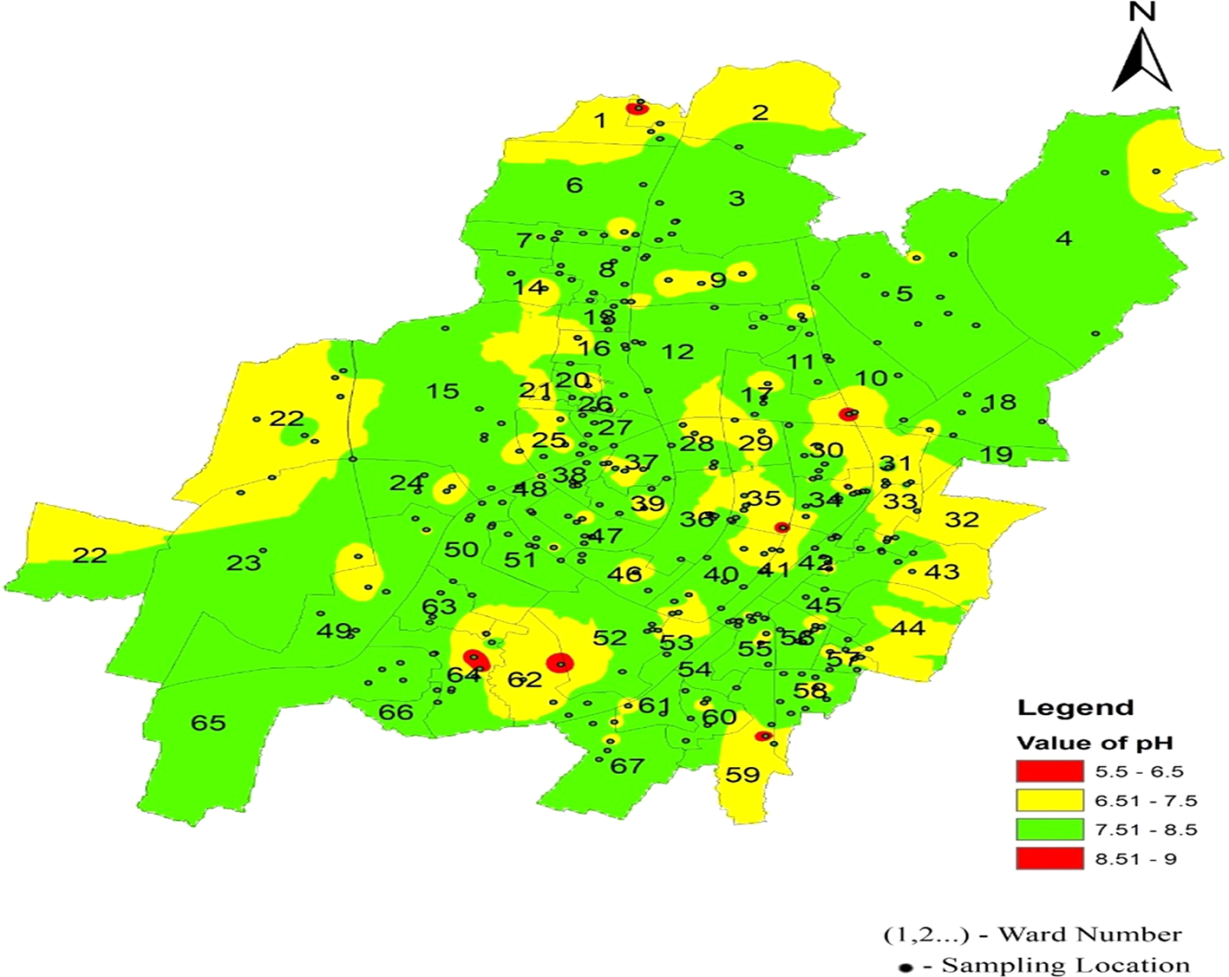
Ward wise pH in supply water of Bhubaneswar (Refer underlying data).

**Figure 8.**
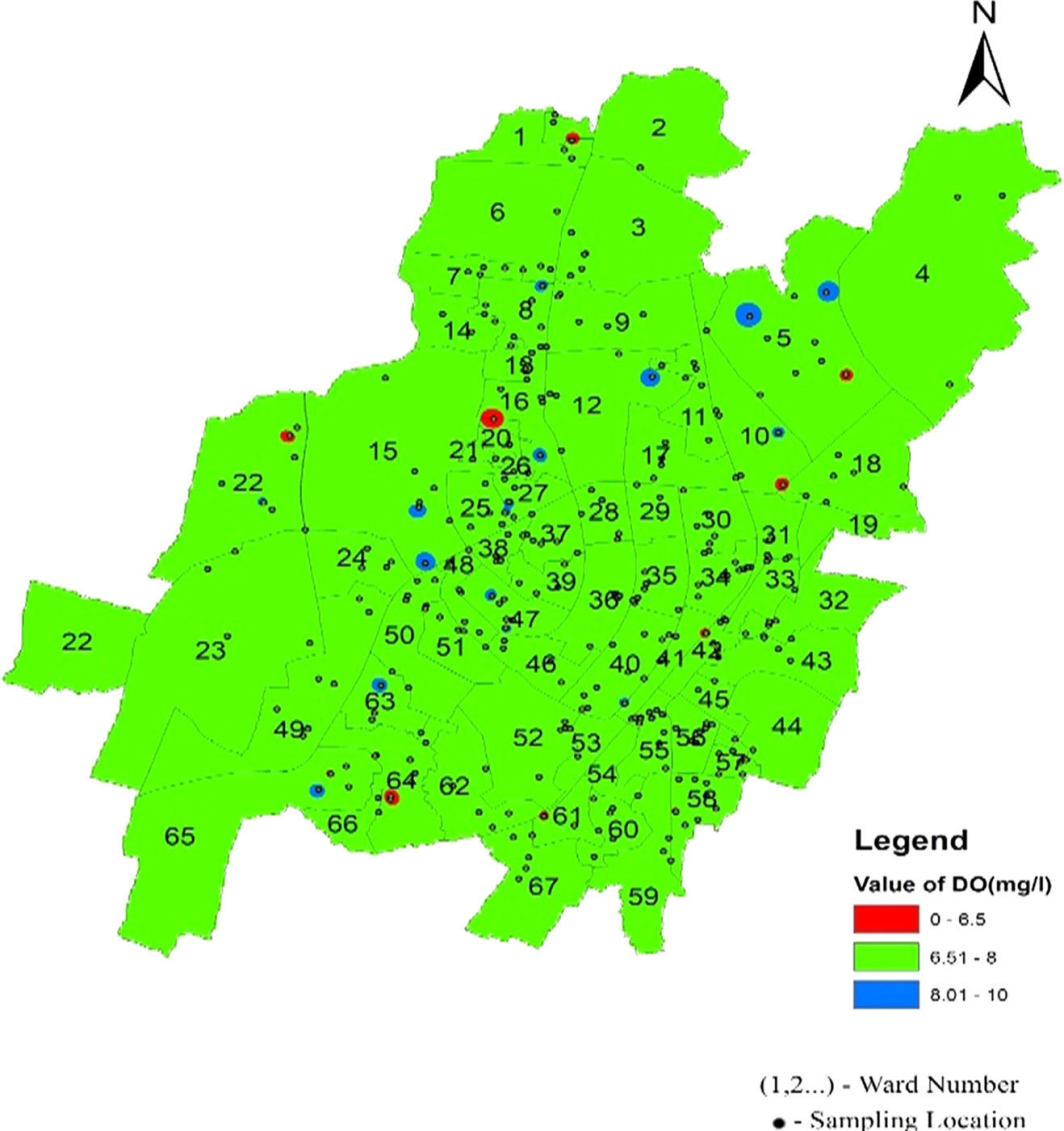
Ward wise dissolved oxygen in supply water of Bhubaneswar.

**Figure 9.**
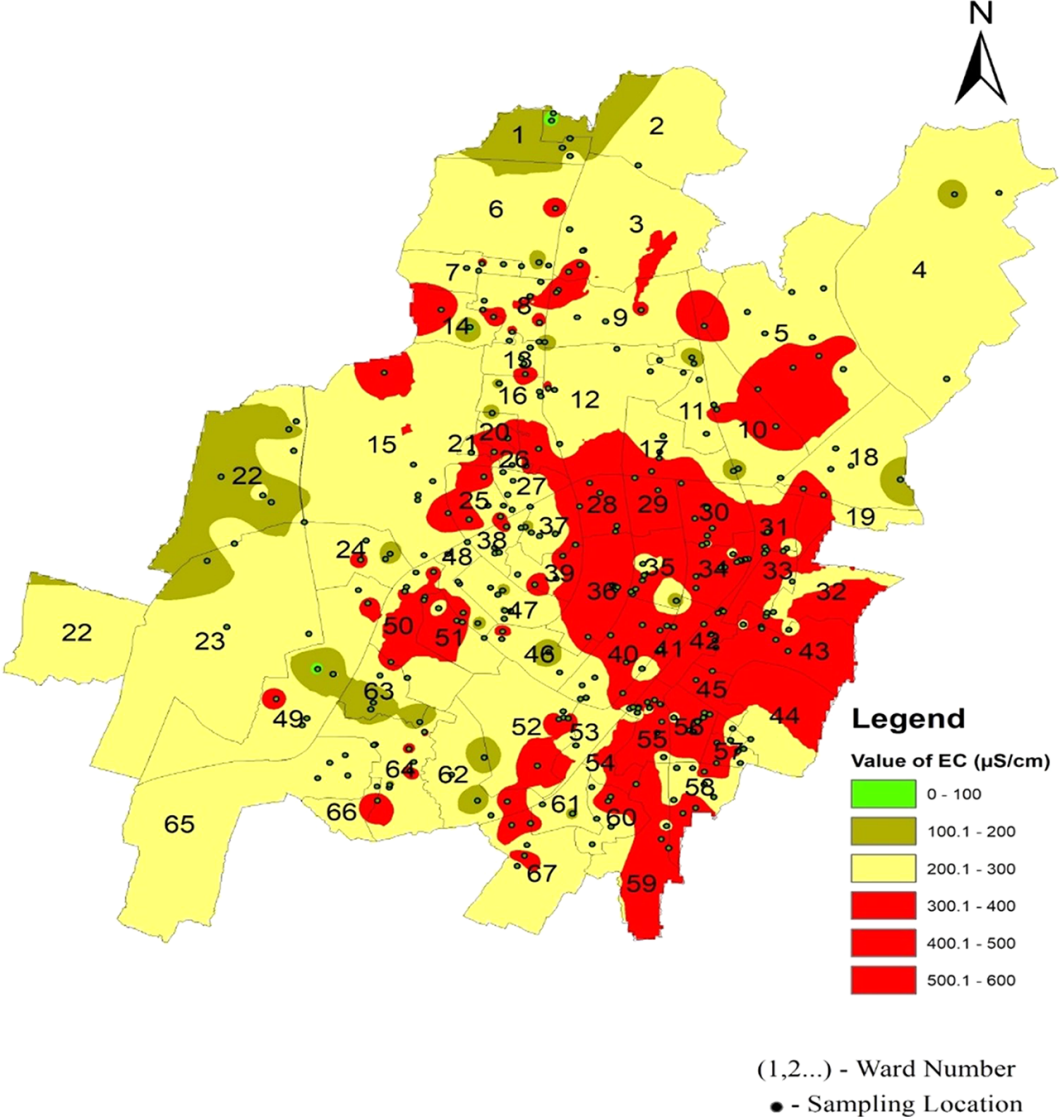
Ward wise electrical conductivity in supply water of Bhubaneswar.

**Figure 10.**
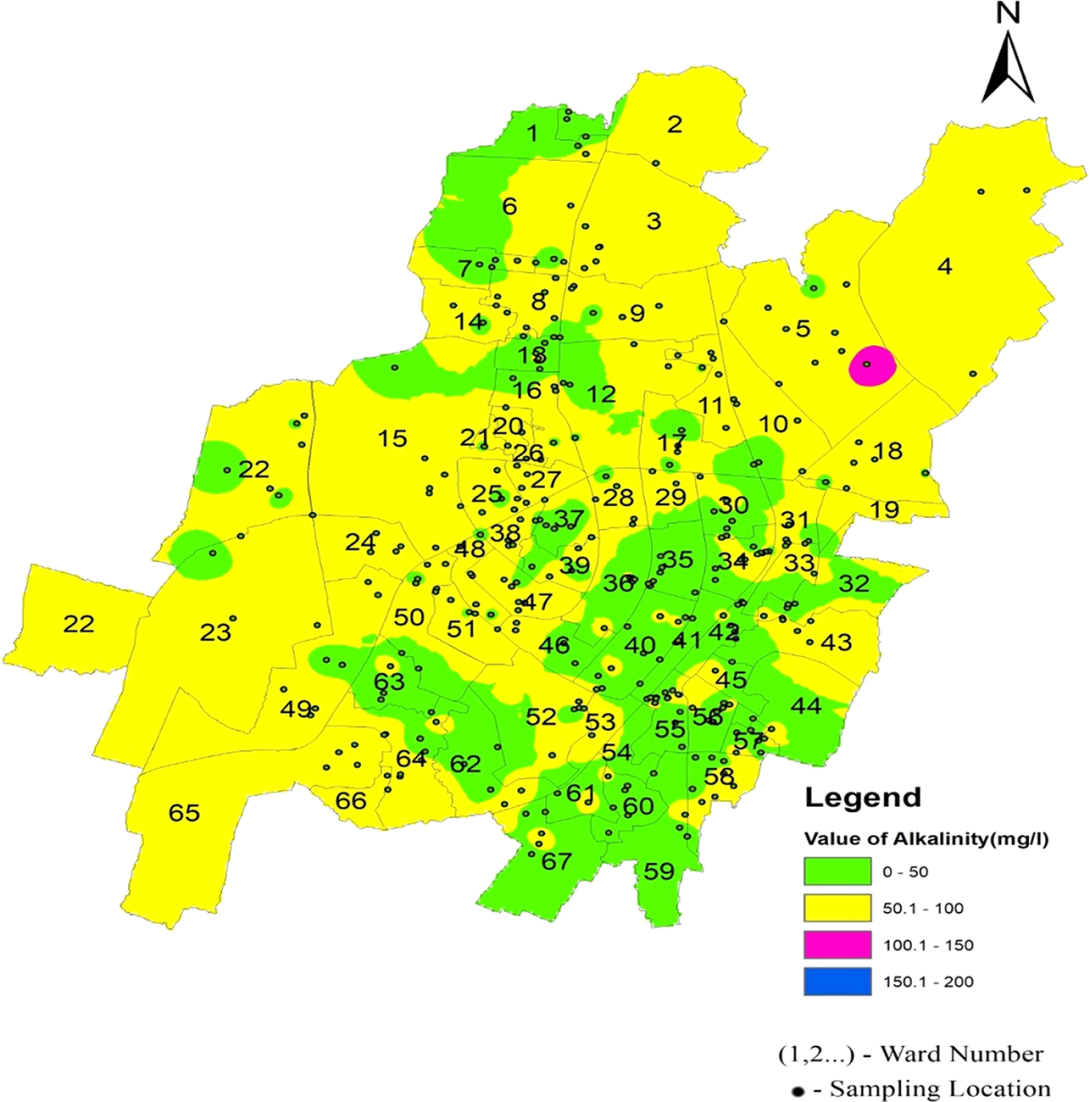
Ward wise alkalinity in supply water of Bhubaneswar.

It is well understood that the electrical conductivity of water determines the minerals and salts that are dissolved in the water. Electrical conductivity ranges from 58.25 s/cm to 480 s/cm in the north zone, 67.7 s/cm to 519.9 s/cm in the South-West zone, and 173 s/cm to 581.47 s/cm in Bhubaneswar’s South-East zone, respectively. As shown in Figure 9 and Figure 6(c), in the north zone, 32.03% of the samples, 27.61% in the south-west zone, and 72% in the south-east zone are beyond the permissible limit. Some areas had a conductivity higher than 500 μs/cm: Sir Pu Bhoi Sahi of ward no. 51, Nico Park Lake area of ward no. 28, Bhoi Sahi of Ward no. 28, Gridco Colony of ward no. 29, Sarala Nagar, and Santoshi Nagar of Ward no. 31, as shown in Figure 9 Ward wise electrical conductivity in supply water of Bhubaneswar Refer underlying data. Runoff water, septic systems, animal waste, and sewage leakage into the ruptured old pipeline system are the causes of this The alkalinity values of the different areas presented in Figure 6(d) indicate that in all three zones, the data are below the permissible limit. Low values of alkalinity were observed in the southeast zone and high values in the northern zone. As shown in Figure 10 (refer underlying data) Ward wise alkalinity in supply water of Bhubaneswar, no areas were affected by the alkalinity.

**Figure 11.**
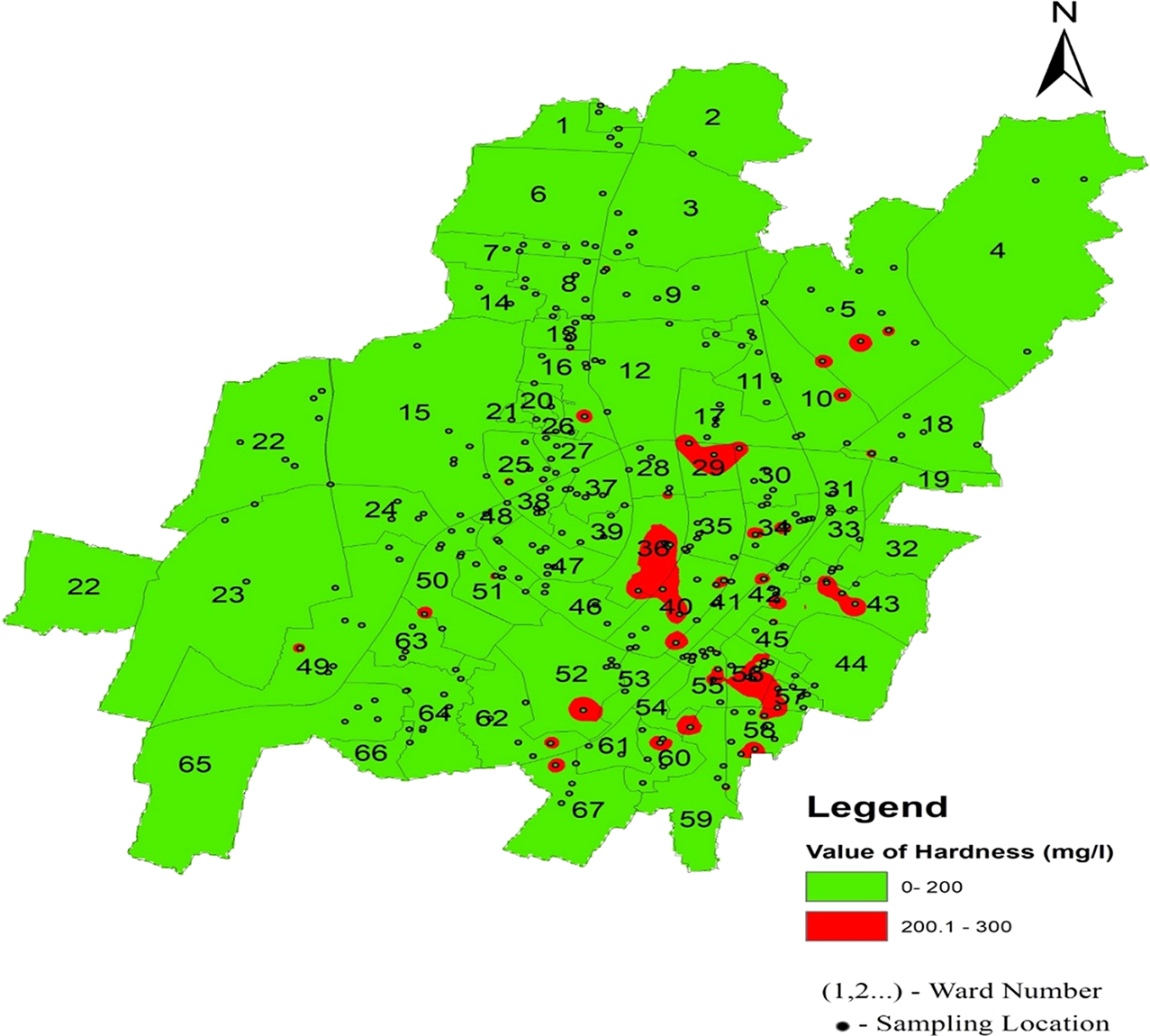
Ward wise hardness in supply water of Bhubaneswar.

Hardness concentration varied between 13.12 mg/l and 262.2 mg/l in the north zone, 13.12 mg/l to 229.6 mg/l in the south-west zone, and 98.4 mg/l to 268.96 mg/l in the south-east zone, with an average value of 118.45 mg/l, 116.96 mg/l, and 186.09 mg/l, respectively, presented in Table 2 and Figure 6(e). The presence of cations such as calcium, magnesium, iron, aluminum, and other bivalent and trivalent cations is the cause of water hardness ( Malakootian et al., 2010). Supply of water with a high hardness level results in a poor taste of water. Furthermore, persistent use of an aberrant concentration in humans might result in kidney stones and cardiovascular issues ( Chaudhary and Satheeshkumar, 2018). The highest deviation in hardness was found in two areas: Champa Pokhari of ward no. 45 and Rabindra Mandap of ward no. 36. These are the maximum values of the hardness of the supply water in Bhubaneswar, as shown in Figure 11 (refer underlying data) Ward wise hardness in supply water of Bhubaneswar.

**Figure 12.**
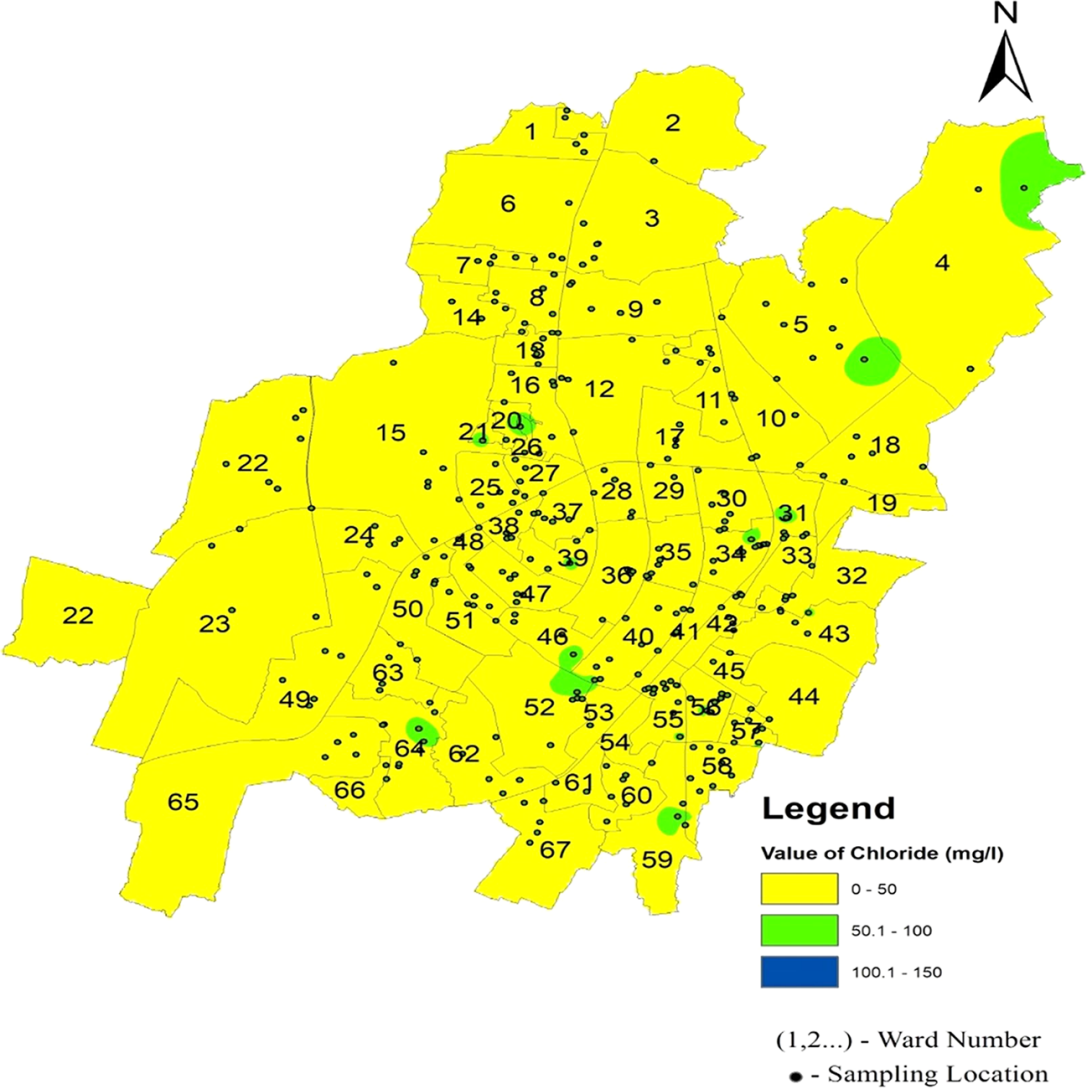
Ward wise chloride in supply water of Bhubaneswar.

Chloride infiltrates supply water systems through a combination of natural processes, such as soil erosion, geological weathering, and human activities, such as fertilizer usage and wastewater discharge ( Clesceri, 2012). In the North zone, the mean chloride level is recorded at 33.86 mg/l, while in the South-West zone, it slightly increases to 34.29 mg/l, and in the South-East zone, it further rises to 38.84 mg/l, as illustrated in Figure 6(f ). The standard deviation values for chloride vary across zones, with the north zone exhibiting 11.65, the South-West zone at 10.76, and the South-East zone at 7.61. These deviations indicate the extent of variability in the chloride levels within each zone. Remarkably, Figure 12 Ward wise chloride in supply water of Bhubaneswar (refer underlying data), shows no deviation from the permissible limit, confirming the safety of the Bhubaneswar water supply in terms of chloride contamination.

**Figure 13.**
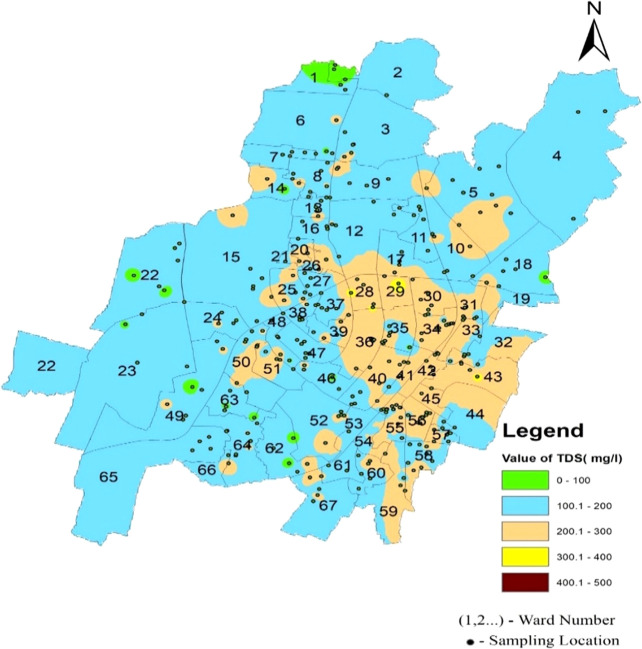
Ward wise Total dissolved solids in supply water of Bhubaneswar.

Total dissolved solids in the supply water varied between 37.86 mg/l and 312.2 mg/l in the north zone, 44.01 mg/l to 337.94 mg/l in the South-West zone, and 112.565 mg/l to 377.96 mg/l in the South-East zone, as per Figure 6(g). The average concentration of the supply water samples of 67 wards of Bhubaneswar was 166.02 mg/l, 163.16 mg/l, and 222.93 mg/l in the above-mentioned three zones, respectively. This demonstrates that the overall quality of the supply water is below the permissible level of 500 mg/l (IS 10500, 2012). In Figure 13 (refer underlying data) Ward wise Total dissolved solids in supply water of Bhubaneswar, it is demonstrated that no total dissolved solid value has exceeded the permissible limit.

**Figure 14.**
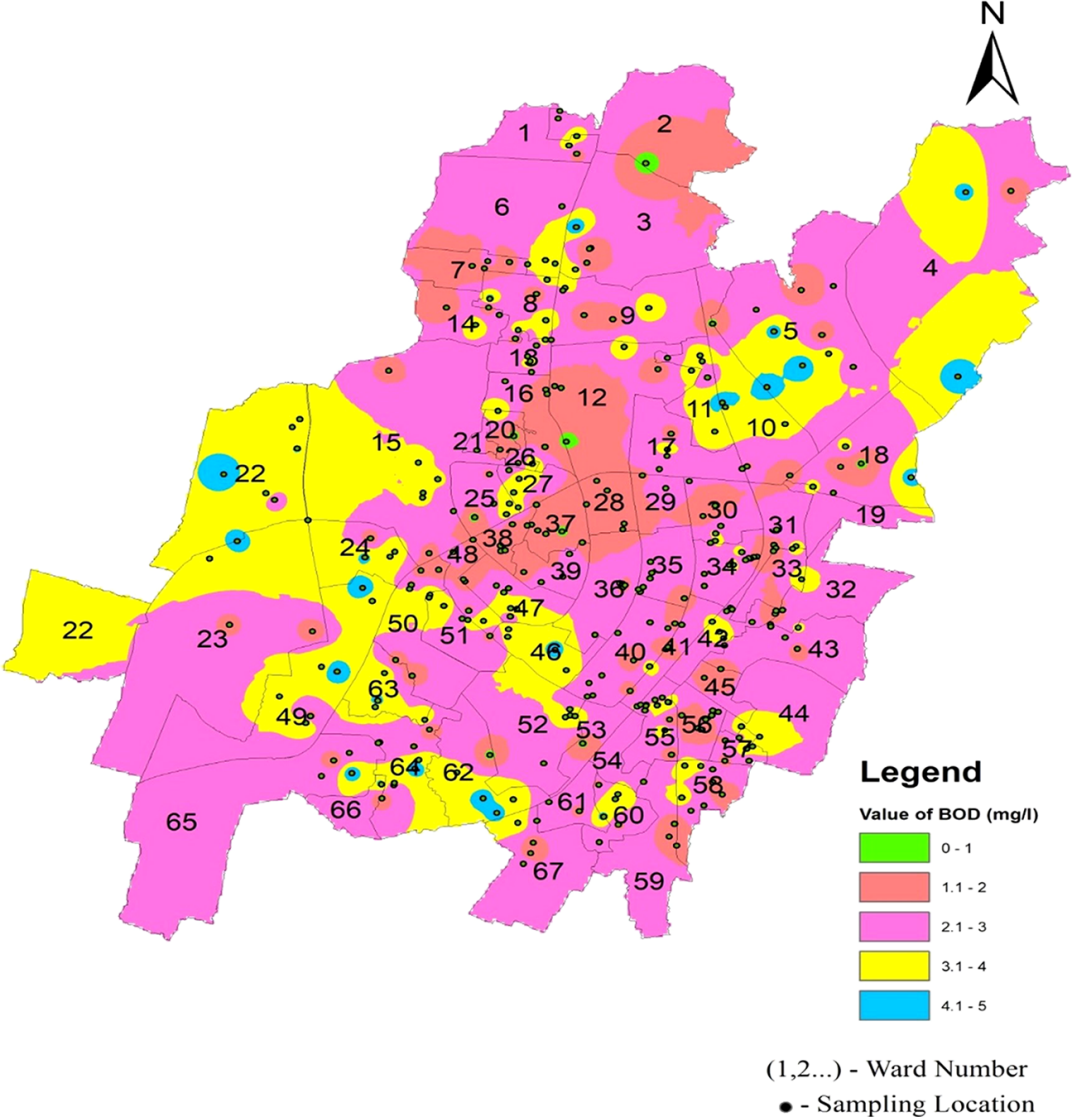
Ward wise biochemical oxygen demand in supply water of Bhubaneswar.

As shown in Figure 6(h), the mean value in the three zones varies from 2.39 2.75. This indicates a relatively consistent range of values across the zones. According to Figure 14 Ward wise biochemical oxygen demand in supply water of Bhubaneswar (refer underlying data), all biochemical oxygen demand values fell within the allowed range. This suggests that the water quality in the measured zones met the specified standards or regulatory requirements, affirming the overall environmental health in those areas.

**Figure 15.**
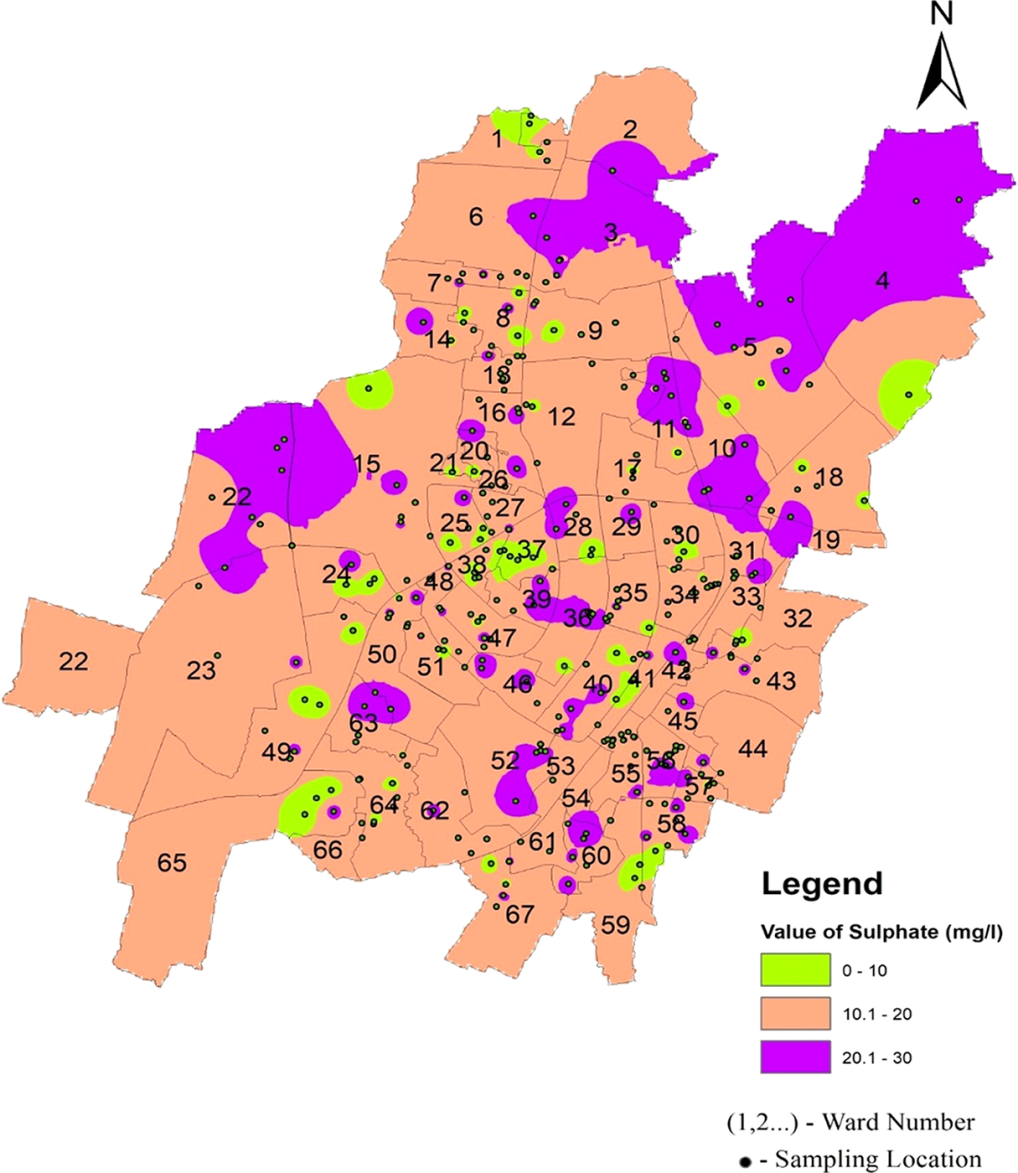
Ward wise sulphate in supply water of Bhubaneswar.

According to IS-10500-2012 standards, the acceptable range for sulfate is set at 200 mg/l, with levels beyond 400 mg/l seeming harmful and potentially disease-causing if used for consumption. Various factors contribute to the presence of sulfate, including mineral dissolution, atmospheric deposition, and human activities such as fertilizer use and mining ( Shengnan et al., 2021). The high sulfate levels found in many global aquifers are largely due to gypsum. The average value ranges between 15.57 mg/l and 17.14 mg/l in the three zones of Bhubaneswar. Figure 15 Ward wise sulphate in supply water of Bhubaneswar (refer underlying data), and Figure 6(i) show that the sulfate value does not exceed the permissible limit.

**Figure 16.**
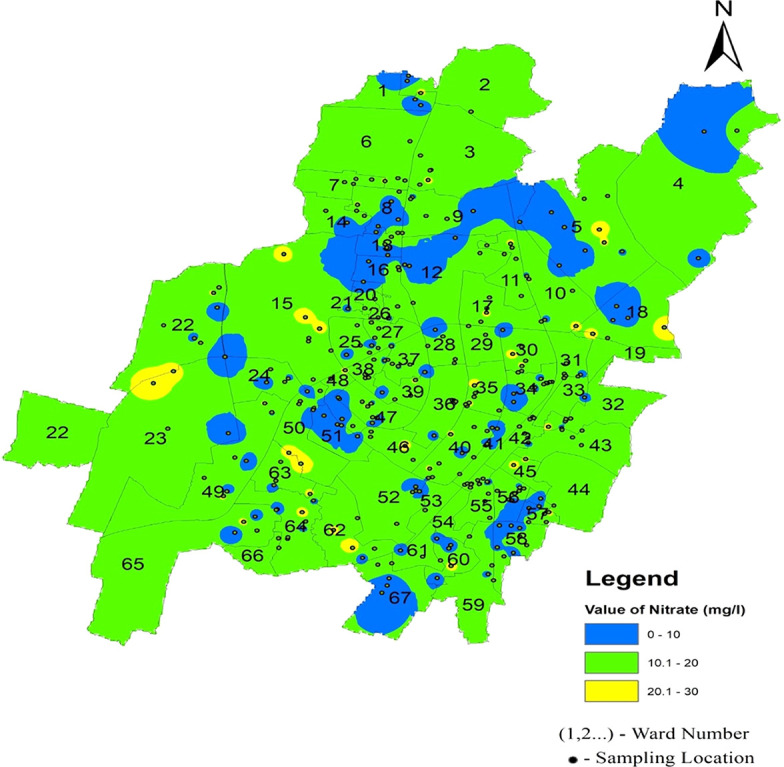
Ward wise nitrate in supply water of Bhubaneswar.

According to IS10500-2012 standards, the nitrate value range was set at 45 mg/l, representing the desired level, and all samples fell within the permissible limit. The maximum observed value was 30 mg/l in both the Southeast and Southwest zones, as illustrated in Figure 6(j). Adherence to regulatory standards is critical for ensuring water safety. The prevalence of high precipitation rates, which facilitate fertilizer infiltration, has been identified as the primary driver behind elevated NO3- values in the source of the supply water ( Wagh et al., 2017, 2018). High concentrations of nitrate cause the blue baby syndrome ( Elwood and van der Werf, 2022). Figure 16 Ward wise nitrate in supply water of Bhubaneswar (refer underlying data), (refer underlying data) indicates that Bhubaneswar supply water is safe in terms of nitrate contamination.

### 3.3 Water Quality Index status values in North zone, South-West zone and South-East zone

From Table 4, it is observed that water quality is excellent and good in the supply water of the northern, south-west, and south-east zones of Bhubaneswar. The highest percentage of excellent water quality was found in the south-east zone of Bhubaneswar (28.58%), while the lowest percentage was found in the south-west zone (9.53%). The highest percentage of good water quality (90.47%) was found in the south-west zone of Bhubaneswar. The northern and south-east zone show 70-80% of a good water quality index. This is because the southeast zone has less human interference. The percentage of good quality is lower in the south-west zone than in others because many old pipeline networks still exist in these areas, as per the report of the Public Health Engineering Department (PHED). No signs of poor, very poor, or unsuitable water quality indices were found in any of the zones.

**Table 4.** Water quality Index status of supply water in different zones of Bhubaneswar ( Gao et al., 2020).

Water Quality Status	North Zone	South-West zone	South-East zone
Excellent Water Quality	23.81%	9.53%	28.58%
Good Water Quality	76.19%	90.47%	71.42%
Poor Water Quality	-	-	-
Very Poor Water Quality	-	-	-
Unsuitable for Drinking	-	-	-

**Figure 17.**
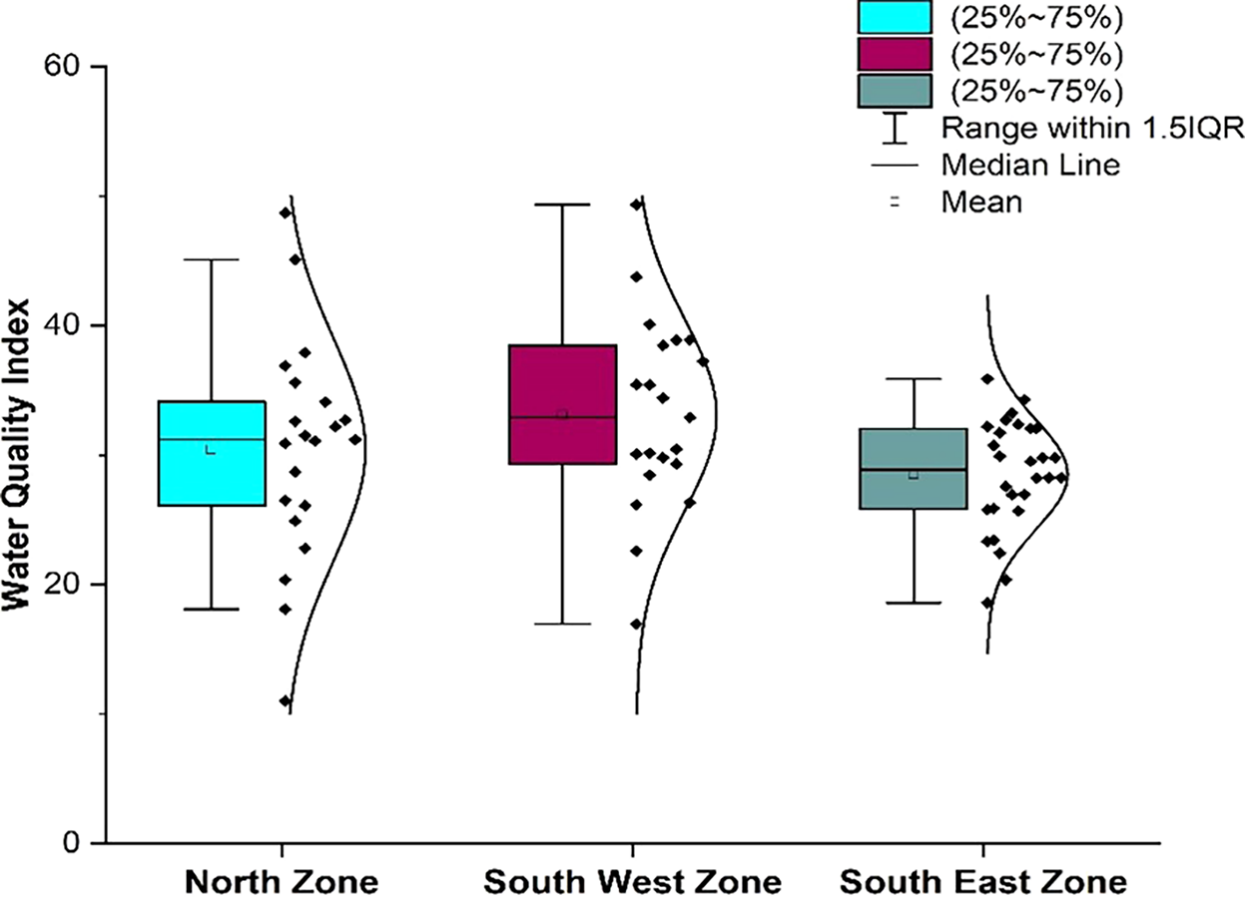
Comparison of water quality index of the north zone, south-west zone and south-east zone.

As shown in Figure 17 Comparison of water Quality Index of supply water in different zones of Bhubaneswar (refer underlying data), the overall water quality of Bhubaneswar is in good condition, although some parameters deviated slightly from their permissible limits. This is because of the large sample size in a greater number of areas, where the deviations in most of the parameters are within the permissible limit, and very few parameters, such as hardness and electrical conductivity, deviated in a slightly higher range from the permissible limit. However, it did not significantly affect the water quality index values.

## 4. Conclusion

This study evaluated the overall water quality of supply water in different zones of Bhubaneswar, where the excellent water quality percentage was comparatively less than the percentage of good water quality. The south-west zone more areas are excellent water quality than north-zone and south-east zone. The study indicates that if the water supply network system is not properly monitored, future water quality deterioration will be a major issue. Suitable policies should be incorporated to address these issues in the future. A pipeline system of higher-grade material can be suggested where quality deterioration has already begun. Advanced technologies for the detection of faults in pipeline systems must be followed prior to water contamination.

## Declarations

### Consent for publication

All authors critically evaluated the manuscript for intellectual content, approved the final version to be published, and agreed to be accountable for all aspects of the work.

## Data Availability

Figshare: Title: Research Raw Data files for supply water of Bhubaneswar, https://doi.org/10.6084/m9.figshare.26819056.v4 ( Samal and Prava 2024). This Project contains the following underlying data:
• Volunerable Area maps using ArcGIS for all the 8 parameters and also Bhubaneswar Study area map (JPG format with 300 dpi) • Water Quality parameters in different zones and it’s comparison (JPG format with 300 dpi). • Excel Data of 10 parameters of 67 wards divided into three zones (.xls) • Excel Data of Water Quality Index calculation of 67 wards divided into three zones (.xls) Data are available under the terms of the Creative Commons Zero ‘No Rights Reserved’ data waiver (CC0 Public domain dedication).
