# Supplementary material for: Assessment of water quality in the piped water supply system by using Water Quality index method
Source: F1000Res. 2025 Oct 30;13:1286. Originally published 2024 Oct 28. [Version 3] doi: 10.12688/f1000research.156276.3 (PMC12618970; doi:10.12688/f1000research.156276.3)
Supplement: Peer review history

# Reviewer report: Reviewer response for version 3

**DOI:** 10.5256/f1000research.188533.r428403

**Published:** 2025-11-14

**Authors:** Majeed Mattar Ramal

**Affiliations:** 1 University of Anbar, Ramadi, Iraq

The revised paper is suitable for indexing

Is the work clearly and accurately presented and does it cite the current literature?

Partly

If applicable, is the statistical analysis and its interpretation appropriate?

Partly

Are all the source data underlying the results available to ensure full reproducibility?

No

Is the study design appropriate and is the work technically sound?

Partly

Are the conclusions drawn adequately supported by the results?

No

Are sufficient details of methods and analysis provided to allow replication by others?

Partly

Reviewer Expertise:

Environmental Engineering, Water Quality , EIA

I confirm that I have read this submission and believe that I have an appropriate level of expertise to confirm that it is of an acceptable scientific standard.

# Reviewer report: Reviewer response for version 3

**DOI:** 10.5256/f1000research.188533.r428405

**Published:** 2025-11-11

**Authors:** Md. Ripaj Uddin

**Affiliations:** 1 Institute of National Analytical Research and Service (INARS), Dhaka, Bangladesh

1. Conclusion may rewrite for better expression.
2. Please update the manuscript by incorporating the relevant literature, such as the following article: https://doi.org/10.1016/j.rsma.2025.104431 ; https://doi.org/10.1016/j.chemosphere.2024.143453 ; https://doi.org/10.1371/journal.pone.0300878 ; https://doi.org/10.1016/j.heliyon.2024.e2785; https://doi.org/10.1016/j.jhazmat.2023.133214

Is the work clearly and accurately presented and does it cite the current literature?

Yes

If applicable, is the statistical analysis and its interpretation appropriate?

Partly

Are all the source data underlying the results available to ensure full reproducibility?

Partly

Is the study design appropriate and is the work technically sound?

Yes

Are the conclusions drawn adequately supported by the results?

Yes

Are sufficient details of methods and analysis provided to allow replication by others?

Partly

Reviewer Expertise:

Environmental Engineering, Water Quality Assessment, Urban Hydrology.

I confirm that I have read this submission and believe that I have an appropriate level of expertise to confirm that it is of an acceptable scientific standard, however I have significant reservations, as outlined above.

# Reviewer report: Reviewer response for version 2

**DOI:** 10.5256/f1000research.178630.r386278

**Published:** 2025-06-16

**Authors:** Eden T. Wasehun

**Affiliations:** 1 North Carolina A&T State University, Greensboro, USA

The manuscript titled ‘ *Assessment of water quality in the piped water supply system by using Water Quality index method*’ has evaluated an important issue of the spatial variation of piped water quality in an Indian smart city using the Weighted Arithmetic Water Quality Index (WAWQI) and GIS based spatial interpolation. This is an important topic to understand piped water quality, but there are some concerns need to improve.

 Abstract:

 Of course, you describe the background, methods you used and results in the abstract, but the problem statement needs more elaboration and concisely description of the sampling design is needed.

 Introduction:

- Although you mentioned that you applied the widely used method, WAWQI, and focused one of the smart city’s piped networks, it is unclear of the explicit contribution of this study. Therefore, suggest to clearly point out the contribution of the study.
- Suggest to cite or add any prior municipal scale WQI studies in comparable Indian cities, and clearly differentiate your work.
- Several statements lack a theoretical basis. It is recommended to cite relevant literature to support the assertations.  For example: Paragraph 4: “The Water Quality Index (WQI), frequently employed by researchers..” needs citation. Paragraph 5: “Few studies on the quality measurement of supply water have assumed that supply water quality deteriorates with the aging of infrastructure..” you cited only one source; either change “few studies” or add more references.

Therefore, consider revising these sentences and some others with appropriate references to strengthen the argument.

 Methods

- It is good you visualize the study area but the map does not show where the each sample was collected. Therefore, suggest to redraw the figure 1a to show the sample locations.
- Why five points per ward, and how were “industries, markets, institutions” prioritized?
- Were samples taken at the same time or day or season across wards? This affects comparability.
- The description of qi and wi is mathematically correct, but the weights used for each parameter is not shown. Therefore, suggest to show the actual weights used in a table.

Result

- The use of percentages of samples exceeding limits is helpful, but consider adding confidence intervals or hypothesis tests when comparing zones.
- Suggest to plot all figures (7- 16) in consistent color scales and legends, to compare the parameters ward wise.
- As can be seen from Table 4 and Figure 17, there is no “poor” or “very poor” zones, but given large parameter deviations, why does the composite WQI remain in the “good” range? A brief sensitivity analysis, for example leaving out one parameter, would help illustrate index robustness.

Discussion

 The paper lacks more deeply interpretation of causes and implications.

- Why does the South-East zone consistently outperform others? Fewer old pipelines or lower vulnerable areas presence?
- What are the study limitations?
- Suggest how the water quality/ environment management/ Minister of Urban Development could use these findings.

Is the work clearly and accurately presented and does it cite the current literature?

Yes

If applicable, is the statistical analysis and its interpretation appropriate?

Yes

Are all the source data underlying the results available to ensure full reproducibility?

Partly

Is the study design appropriate and is the work technically sound?

Yes

Are the conclusions drawn adequately supported by the results?

Yes

Are sufficient details of methods and analysis provided to allow replication by others?

Partly

Reviewer Expertise:

Water quality, remote sensing, machine learning and deep learning application for environmental science

I confirm that I have read this submission and believe that I have an appropriate level of expertise to confirm that it is of an acceptable scientific standard, however I have significant reservations, as outlined above.

# Reviewer report: Reviewer response for version 2

**DOI:** 10.5256/f1000research.178630.r386283

**Published:** 2025-05-28

**Authors:** Md. Ripaj Uddin

**Affiliations:** 1 Institute of National Analytical Research and Service (INARS), Dhaka, Bangladesh

Dear authors,

 The manuscript titled "Assessment of water quality in the piped water supply system by using Water Quality Index method" presents a comprehensive evaluation of water quality in Bhubaneswar’s piped supply system. The study aligns with SDG 6 and employs a robust methodology, including spatial analysis via ArcGIS and the Weighted Arithmetic Water Quality Index (WQI). While the work is valuable, certain areas require clarification and improvement to enhance its scientific rigor and readability.

 1. **Abstract:** The problem statement and significance of deviations (e.g., 43.88% in conductivity) should be emphasized more clearly.

 2. **Introduction:** The novelty of the study (e.g., focus on piped systems vs. groundwater) needs stronger justification. Link more explicitly to prior literature on urban water supply challenges.

 3. **Methodology**

- Clarify whether samples were collected at consistent times/seasons to account for temporal variability.
- The absence of microbial testing (e.g., *E. coli*) is a significant omission for a drinking water study. Justify or address this limitation.
- Update references to APHA (1926) with the latest edition (e.g., APHA 2024).

4. **Results and Discussion**

- Include basic statistical tests (e.g., ANOVA) to validate zone-wise differences in parameters like pH and conductivity.
- Add scales and legends to Figures 7–16 for better interpretability.
- Compare results with similar studies in other Indian cities (e.g., Kolkata, Indore) to contextualize findings.
- Incorporate indices like Langelier or Ryznar to assess pipeline corrosion risks.
- Suggest specific strategies for community engagement (e.g., workshops, mobile apps for reporting leaks).
- Correct Table 2 (e.g., EC should be µS/cm, not mg/l).
- Improve resolution and label clarity in maps (e.g., ward numbers in Figures 7–16).
- Ensure all data (e.g., raw parameter values) are accessible via Figshare or supplementary files.
- Consider a follow-up study incorporating microbial testing and advanced statistical modeling (e.g., PCA).

5. **References:** Expand citations to include recent WQI studies (e.g., Tyagi et al., 2013) and urban water quality literature.

Is the work clearly and accurately presented and does it cite the current literature?

Yes

If applicable, is the statistical analysis and its interpretation appropriate?

Partly

Are all the source data underlying the results available to ensure full reproducibility?

Partly

Is the study design appropriate and is the work technically sound?

Yes

Are the conclusions drawn adequately supported by the results?

Yes

Are sufficient details of methods and analysis provided to allow replication by others?

Partly

Reviewer Expertise:

Environmental Engineering, Water Quality Assessment, Urban Hydrology.

I confirm that I have read this submission and believe that I have an appropriate level of expertise to confirm that it is of an acceptable scientific standard, however I have significant reservations, as outlined above.

# Reviewer report: Reviewer response for version 2

**DOI:** 10.5256/f1000research.178630.r382667

**Published:** 2025-05-20

**Authors:** Majeed Mattar Ramal

**Affiliations:** 1 University of Anbar, Ramadi, Iraq

The study evaluates the water quality of Bhubaneswar’s piped supply system using the Weighted Arithmetic Water Quality Index (WQI) method. It aligns with SDG 6 (clean water and sanitation) and addresses gaps in monitoring urban water distribution networks. The methodology involves sampling 335 locations across 67 wards, analyzing 10 physicochemical parameters, and mapping spatial variations via ArcGIS. Key findings indicate overall "good" to "excellent" water quality, with localized deviations in pH, conductivity, and hardness linked to aging infrastructure and construction activities.

**Overall Evaluation**

- **Originality**: Focus on urban piped systems adds value vs. groundwater/river studies.
- **Impact**: Findings aid Bhubaneswar’s PHED in infrastructure planning; model applicable to similar cities.
- **Revisions Needed**: Address methodological gaps (bacteriological tests, statistical depth) and improve clarity.

**Recommendation**: **Accept with Minor Revisions**

Is the work clearly and accurately presented and does it cite the current literature?

Partly

If applicable, is the statistical analysis and its interpretation appropriate?

Partly

Are all the source data underlying the results available to ensure full reproducibility?

No

Is the study design appropriate and is the work technically sound?

Partly

Are the conclusions drawn adequately supported by the results?

No

Are sufficient details of methods and analysis provided to allow replication by others?

Partly

Reviewer Expertise:

Environmental Engineering, Water Quality , EIA

I confirm that I have read this submission and believe that I have an appropriate level of expertise to confirm that it is of an acceptable scientific standard, however I have significant reservations, as outlined above.

# Response

**Published:** 2025-09-08

**Authors:** Kshyana Prava Samal

Reviewer 4

 The study evaluates the water quality of Bhubaneswar’s piped supply system using the Weighted Arithmetic Water Quality Index (WQI) method. It aligns with SDG 6 (clean water and sanitation) and addresses gaps in monitoring urban water distribution networks. The methodology involves sampling 335 locations across 67 wards, analyzing 10 physicochemical parameters, and mapping spatial variations via ArcGIS. Key findings indicate overall "good" to "excellent" water quality, with localized deviations in pH, conductivity, and hardness linked to aging infrastructure and construction activities.

**Overall Evaluation**

- **Originality**: Focus on urban piped systems adds value vs. groundwater/river studies.
- **Impact**: Findings aid Bhubaneswar’s PHED in infrastructure planning; model applicable to similar cities.
- **Revisions Needed**: Address methodological gaps (bacteriological tests, statistical depth) and improve clarity.

**Recommendation**: **Accept with Minor Revisions**

- **Is the work clearly and accurately presented and does it cite the current literature?**

Partly

- **Ans:**
- **Alignment with Research Objectives:** The chosen design allows for the precise measurement of all  the primary physico chemical parameters in the laboratory and scientifically validated with Govt laboratory checking . We

**2. Technical Soundness of the Work:**

- **Methodological Rigor:** All experimental protocols, data collection procedures, and analytical techniques were taken care using the, APHA, 2022. Where applicable, standard operating procedures (SOPs) were followed.
- **Data Quality and Reproducibility:** Steps were taken to ensure data integrity. Sample sizes were justified to ensure the study had sufficient statistical power to detect a meaningful effect.
- **Statistical Analysis:** The statistical methods are appropriate for the type of data collected. We have consulted with professional statisticians to confirm the correctness of our approach, which includes water quality analysis,  Arc Gis for mapping the volunerable areas and for multiple comparisons were applied where  it is necessary.

 

- **Is the study design appropriate and is the work technically sound?**
- Partly

Ans:

 We thank the reviewer for raising this critical point. We are confident that the study design is robust and appropriate for addressing our research objectives, and that the work is technically sound.

**Study Design:** We collected 335 pipe water samples from 67 wards, tested and analyzed them in the State Pollution Control Board laboratory following IS 10500:2012 standards. All protocols are well-established, with detailed descriptions provided in the Methods section to ensure transparency and replicability.

- **If applicable, is the statistical analysis and its interpretation appropriate?**

Partly

**Ans: Statistical Analysis:** The statistical methods were appropriately chosen for the data structure and align with widely accepted applications of the water quality index reported in previous studies.

  

- **Are all the source data underlying the results available to ensure full reproducibility?**

No

 Yes, we are committed to open science and reproducibility. All source data underlying our results are publicly available in **Figshare**: *Research Raw Data files for supply water of Bhubaneswar* (Samal and Prava, 2024), DOI: https://doi.org/10.6084/m9.figshare.26819056.v4.

**Data Availability Statement (for manuscript):**

 All data generated or analyzed during this study are included in this article and its supplementary files, and are also available at the above repository.

- **Are the conclusions drawn adequately supported by the results?**

No

**Ans:** We thank the reviewer for this important question. We believe our conclusions are robust and directly supported by the results. The water quality index across wards and parameters beyond permissible limits are carefully presented in the results section.

1. **Direct Data–Claim Linkage:** Each conclusion is explicitly supported by figures and tables, with evidence clearly shown in box plots and ArcGIS maps.
2. **Statistical Support:** All reported differences and effects are backed by appropriate statistical analyses.

Within the defined scope of this study, we are confident that the results provide a solid and sufficient foundation for our conclusions, with a clear and logical evidence-based presentation.

 Competing Interests

*No competing interests were disclosed.*

 Reviewer Expertise

 Environmental Engineering, Water Quality , EIA

**I confirm that I have read this submission and believe that I have an appropriate level of expertise to confirm that it is of an acceptable scientific standard, however I have significant reservations, as outlined above.**

# Reviewer report: Reviewer response for version 1

**DOI:** 10.5256/f1000research.171563.r350892

**Published:** 2025-01-13

**Authors:** Sudhir K Srivastava

**Affiliations:** 1 Central Ground Water Board, Lucknow, UP, India

The authors have reported the analysis of Fe+2 and Zn ^2+^ under Introduction para 8th, but no where the data of  Fe+2 has been seen or utilized. APHA (1926) is very old version of the book the latest version is 2023 edition.

 The most of the chemical analysis has been done on out put point of piped supply, not the source point of water supply. so the BOD values  are not appropriate as the supply water has travelled so much distance. It has not been cleared about the treatment of surface water before its supply.

Is the work clearly and accurately presented and does it cite the current literature?

Yes

If applicable, is the statistical analysis and its interpretation appropriate?

Partly

Are all the source data underlying the results available to ensure full reproducibility?

Partly

Is the study design appropriate and is the work technically sound?

Yes

Are the conclusions drawn adequately supported by the results?

Partly

Are sufficient details of methods and analysis provided to allow replication by others?

Partly

Reviewer Expertise:

Water Quality Ground Water Quality and Hydrochemistry

I confirm that I have read this submission and believe that I have an appropriate level of expertise to confirm that it is of an acceptable scientific standard, however I have significant reservations, as outlined above.

## References

[1] : VULNERABILITY TO CONTAMINATION OF SHALLOW GROUNDWATER OF BHUBANESWAR CITY DUE TO URBANIZATION. *Research gate:* .2000;

[2] : Contamination of shallow groundwater of Bhubaneswar city due to urbanization. *Research gate:* .2014;

# Response

**Published:** 2025-02-20

**Authors:** Kshyana Prava Samal

Thank you, Sir, for your valuable feedback and suggestions to improve the manuscript.

 The authors have reported the analysis of Fe+2 and Zn ^2+^ under Introduction para 8th, but no where the data of  Fe+2 has been seen or utilized. APHA (1926) is very old version of the book the latest version is 2023 edition.

**Ans** :

 The objective of the project is to analyze the physico-chemical parameters of supply water in the piped water supply system. Consequently, the Fe+2 and Zn2+ parameters related to the piped water supply system have been reviewed in Section 8 of the introduction. Since the scope of the project was limited to physico-chemical parameters, testing for Fe+2 and Zn2+ was not conducted. We greatly appreciate your comments, and it will be taken into consideration in the next phase of the project.

 The most of the chemical analysis has been done on out put point of piped supply, not the source point of water supply. so the BOD values  are not appropriate as the supply water has travelled so much distance. It has not been cleared about the treatment of surface water before its supply.

**Ans:**

 Most of the chemical analysis has been conducted at the output point of the pipe water supply, as the water in the pipeline system comes from the water treatment plant. The BOD values at the source point have not been considered, as the water is treated before distribution and meets the required standard limits.

- **Is the work clearly and accurately presented and does it cite the current literature?**

Yes

- **Is the study design appropriate and is the work technically sound?**

Yes

- **Are sufficient details of methods and analysis provided to allow replication by others?**

Partly

- **If applicable, is the statistical analysis and its interpretation appropriate?**

Partly

- **Are all the source data underlying the results available to ensure full reproducibility?**

Partly

- **Are the conclusions drawn adequately supported by the results?**

Partly

# Reviewer report: Reviewer response for version 1

**DOI:** 10.5256/f1000research.171563.r348678

**Published:** 2025-01-13

**Authors:** Bouselsal Boualem

**Affiliations:** 1 University of Kasdi Merbah, Ouargla, Algeria

First of all, thank you for inviting me to review this article  "Assessment of water quality in the piped water supply system by using Water Quality Index method". After reading and analyzing it, here are my main observations:

**Content of the Article**

 The article analyzes the quality of drinking water in Bhubaneswar, India, using the Water Quality Index (WQI). This study aligns with the Sustainable Development Goals (SDGs), particularly SDG 6, which aims to ensure universal access to safe and high-quality drinking water. The researchers collected and analyzed 335 water samples from three zones of the city (north, south-west, and south-east), covering 67 neighborhoods. Parameters such as pH, dissolved oxygen, electrical conductivity, and hardness were evaluated in accordance with WHO and Bureau of Indian Standards (BIS) guidelines. The results reveal that the water quality is generally good to excellent, with the south-east zone showing the best performance due to minimal human interference. However, some deviations from standards were observed, particularly in terms of pH and conductivity, mainly in areas with aging infrastructure or ongoing construction work. The authors recommend infrastructure improvements, enhanced monitoring, and the use of advanced technologies to prevent future water quality deterioration.

**Strengths of the Article**

 The article stands out for its representative sampling, encompassing 335 samples from 67 neighborhoods, and its use of the Water Quality Index (WQI), which facilitates a comprehensive and understandable evaluation. It highlights disparities between the studied zones and identifies causes of degradation, such as aging infrastructure. Aligned with the Sustainable Development Goals, the study proposes practical recommendations, including pipeline upgrades and the use of advanced technologies. Its rigorous methodology, adherence to international standards, and transparent data presentation make it a valuable contribution to urban water quality management.

**Recommendations for the Authors**

**1. Abstract**

- The abstract should clarify the problem addressed by the study, its significance, and the method employed.
- Key findings should be highlighted concisely and quantitatively.
- For example, specifying the parameters with significant deviations and the percentage of affected zones would strengthen the impact of the results.

**2. Introduction**

- The novelty and specific contributions of the study should be clearly described and justified.
- Briefly compare the advantages of the proposed approach, such as the Water Quality Index (WQI), with existing methods.
- Although the study is specific to Bhubaneswar, demonstrate how its findings can be applied to other regions with similar contexts (e.g., rapid urbanization, aging infrastructure).
- Clarify the study's objectives and present them clearly at the end of the introduction.

**3. Methodology**

- The section "2.1 Study Area" should be limited to describing the geographical, social, and economic characteristics of the study area. Subsections on sampling and mapping should be separated for better clarity.
- The study does not address the bacteriological quality of water, a critical aspect for detecting contamination from wastewater, which poses a significant health risk.

**4. Results and Discussion**

- Present the chemical composition of reservoir water before its injection into the network to analyze changes during distribution.
- Describe the water treatment methods before distribution, as these can influence parameters such as hardness and chlorine concentration.
- Compare the findings with guideline values from the literature (Sawyer and McCarty, 1967; USEPA, 1994 for hardness; APHA, 2005 for TDS and electrical conductivity). Include scatter plots (e.g., TDS vs hardness) to highlight relationships between parameters.
- Add relevant indices to detect risks of scaling and corrosion in the distribution network (e.g., Langelier, Ryznar, Larson-Skold indices).
- Use a Piper diagram to determine the chemical facies of water and analyze its evolution within the network.
- Improve maps by adding scales and locality names for better readability.

**5. Conclusion**

- Include specific and practical recommendations for managing water distribution networks, such as modernizing infrastructure and using corrosion-resistant materials.
- Propose targeted policy interventions to ensure consistent drinking water quality, especially in regions facing similar challenges.

**6. References**

- The number of cited references is insufficient. More high-quality articles should be consulted, particularly on water quality assessment methods and risks related to chemical quality.

Is the work clearly and accurately presented and does it cite the current literature?

Yes

If applicable, is the statistical analysis and its interpretation appropriate?

Yes

Are all the source data underlying the results available to ensure full reproducibility?

Yes

Is the study design appropriate and is the work technically sound?

Yes

Are the conclusions drawn adequately supported by the results?

Yes

Are sufficient details of methods and analysis provided to allow replication by others?

Partly

Reviewer Expertise:

Fundamental Hydrogeology, Hydrogeochemistry, Water Drilling and Hydrodynamics, Groundwater Vulnerability and Management, Water Pollution, Hydrogeological Mapping, Aquifer Recharge and Potential Assessment.

I confirm that I have read this submission and believe that I have an appropriate level of expertise to confirm that it is of an acceptable scientific standard, however I have significant reservations, as outlined above.

# Response

**Published:** 2025-02-20

**Authors:** Kshyana Prava Samal

**Reviewer 3**

 First of all, thank you for inviting me to review this article  "Assessment of water quality in the piped water supply system by using Water Quality Index method". After reading and analyzing it, here are my main observations:

**Content of the Article**

 The article analyzes the quality of drinking water in Bhubaneswar, India, using the Water Quality Index (WQI). This study aligns with the Sustainable Development Goals (SDGs), particularly SDG 6, which aims to ensure universal access to safe and high-quality drinking water. The researchers collected and analyzed 335 water samples from three zones of the city (north, south-west, and south-east), covering 67 neighborhoods. Parameters such as pH, dissolved oxygen, electrical conductivity, and hardness were evaluated in accordance with WHO and Bureau of Indian Standards (BIS) guidelines. The results reveal that the water quality is generally good to excellent, with the south-east zone showing the best performance due to minimal human interference. However, some deviations from standards were observed, particularly in terms of pH and conductivity, mainly in areas with aging infrastructure or ongoing construction work. The authors recommend infrastructure improvements, enhanced monitoring, and the use of advanced technologies to prevent future water quality deterioration.

**Strengths of the Article**

 The article stands out for its representative sampling, encompassing 335 samples from 67 neighborhoods, and its use of the Water Quality Index (WQI), which facilitates a comprehensive and understandable evaluation. It highlights disparities between the studied zones and identifies causes of degradation, such as aging infrastructure. Aligned with the Sustainable Development Goals, the study proposes practical recommendations, including pipeline upgrades and the use of advanced technologies. Its rigorous methodology, adherence to international standards, and transparent data presentation make it a valuable contribution to urban water quality management.

**Recommendations for the Authors**

**1. Abstract**

- The abstract should clarify the problem addressed by the study, its significance, and the method employed.

Ans:

 Provided full length of the abstract for your kind reference.

  

- Key findings should be highlighted concisely and quantitatively.

Ans:

 Provided full length of the abstract for your kind reference.

  

- For example, specifying the parameters with significant deviations and the percentage of affected zones would strengthen the impact of the results.

Ans:

 As per the journal format the abstract is categorized into three parts such as background, methods and results. All of three sections are included under the abstract section.

**2. Introduction**

- The novelty and specific contributions of the study should be clearly described and justified.

Ans:

 The objective of the project was to analysephysico-chemical parameter of water quality. The finding of the study shall help policy makers to develop a policy for regular monitoring and take necessary steps to replace pipe line system in phase wise  manner.

- Briefly compare the advantages of the proposed approach, such as the Water Quality Index (WQI), with existing methods.

Ans:

 The methods like National Sanitation Foundation Water Quality Index (NSFWQI), Canadian Council of Ministers of the Environment Water Quality Index(CCME WQI), Oregon Water Quality Index (OWQI) have been compared with Weighted Arithmetic Water Quality Index Method by Shweta Tyagi(2013) and Weighted Arithmetic Water Quality Index Method was   found suitable among all.

 Tyagi, S., Sharma, B., Singh, P., & Dobhal, R. (2013). Water quality assessment in terms of water quality index. American Journal of water resources, 1(3), 34-38.

  

- Although the study is specific to Bhubaneswar, demonstrate how its findings can be applied to other regions with similar contexts (e.g., rapid urbanization, aging infrastructure).

The similar cities having old age pipeline system, needs to monitor water quality of physico-chemical parameters periodically. If there is deterioration of water quality, operators and policy makers to take necessary steps as suggested in conclusion paragraph.

- Clarify the study's objectives and present them clearly at the end of the introduction.

Ans:

 Given in abstract and introduction section.

**3. Methodology**

- The section "2.1 Study Area" should be limited to describing the geographical, social, and economic characteristics of the study area. Subsections on sampling and mapping should be separated for better clarity.

Ans:

 Agree. However, the section is concise to depict all the geographical, social, economic along sampling process into one section for better understanding.

- The study does not address the bacteriological quality of water, a critical aspect for detecting contamination from wastewater, which poses a significant health risk.

The suggested area may be taken up in the next phase of the project.

**4. Results and Discussion**

- Present the chemical composition of reservoir water before its injection into the network to analyze changes during distribution.

The scope of the project was to analyse water quality parameters within the distribution system. It is understood from the department that raw water treated at a treatment plant to a desire standard which is situated at Mundali (lat-  long- ) on  bank of Mahanandi river. Therefore, it is not necessary to compare reservoir water quality with distribution network water quality.

- Describe the water treatment methods before distribution, as these can influence parameters such as hardness and chlorine concentration.

It is understood from the department that raw water treated at a conventional water treatment plant to a desire standard which is situated at Mundali (lat-  long- ) on bank of Mahanandi river

- Compare the findings with guideline values from the literature (Sawyer and McCarty, 1967; USEPA, 1994 for hardness; APHA, 2005 for TDS and electrical conductivity). Include scatter plots (e.g., TDS vs hardness) to highlight relationships between parameters.

Ans:

 The average hardness value of each sampled zone has been compared with the standard mentioned by Sawyer and McCarty, 1967 and USEPA, 1994. It is found that piped water supply is in the range of moderately high hardness. The comparison statement is shown in below table 1.0

 Table 1.0 Comparison of hardness parameter

 Sample Zone

 Field Observation (Average Value)

 Total Hardness as CaCO3 (mg/l)

 Classification as per (Sawyer and McCarthy 1967)

 Classification as per USEPA, 1994

 North

 118.45

 Moderately high

  moderately hard

 South-West

 116.96

 Moderately high

  moderately hard

 South-East

 186.09

 Hard

 very hard

 TDS and Electrical conductivity have been tested referring APHA 2022 standard method. The scatter plot between TDS and hardness has been generated and shown in Fig 1.0 to Fig 3.0.

 Fig 1.0 Scatter plot for TDS and Hardness minimum value - Reference link: *https://f1000research.s3.amazonaws.com/linked/711235.Figure_1.0.pdf*

 Fig 2.0 Scatter plot for TDS and Hardness maximum value - Reference link: *https://f1000research.s3.amazonaws.com/linked/711236.Figure_2.0.pdf*

 Fig 3.0 Scatter plot for TDS and Hardness average value - Reference link: *https://f1000research.s3.amazonaws.com/linked/711237.Figure_3.0.pdf*

- Add relevant indices to detect risks of scaling and corrosion in the distribution network (e.g., Langelier, Ryznar, Larson-Skold indices).

The department is taking necessary steps to replace the old pipes In phase manner. The scope of the study was to analyse water quality parameter and detect iron level which possibly due to corrosion. Therefore, specific importance to scaling and corrosion has not been stated.

# Reviewer report: Reviewer response for version 1

**DOI:** 10.5256/f1000research.171563.r338598

**Published:** 2025-01-07

**Authors:** Taj Muhammad Jahangir

**Affiliations:** 1 University of Sindh, Jamshoro, Sindh, Pakistan

I am writing the following comments for the improvement of manuscript;

1. The manuscript is short and may be short communication. Overall each section needs improvements.
2. The real objectives may given for to address within the introduction.
3. Those tests conducted in present work, may only be basis of objectives.
4. Table 2 guideline of EC is not mg/l, check other units and limits.
5. The nitrate 30 mg/l is quite high in rivers, It may checked, the source of pollution in ice melt water.
6. The source of water at within rivers that did not compared before wards.
7. Inter river comparison may also present of three rivers.
8. The methodology of parameters i.e nitrate may also explained. Therefore WQI within good quality is suspected and may checked.
9. The results of analysis may compared for statistical analysis, i.e t-test, PCA, Hierarchal analysis, piper diagram, SAR for irrigation.
10. The table for physico chemical parameters may also given supplementary.
11. The discussion is very short and the number parameters were also less included, i.e metals.
12. The abstract and conclusion should also improved.
13. The results were not compared with local studies, and international rivers or water supply.
14. Importance of water if any public water, dependency of type of people on supply water that did not given in detail.
15. The nature of water in different wards may  compared with other smart cities.
16. Why samples crossed the limits, what was the reasons and how public awareness. Indicate the samples with GPS and contaminated samples.
17. Indicate the mass balance and error.
18. The spatial variation may also explained.
19. Figures may improved with ranges to better view.

Is the work clearly and accurately presented and does it cite the current literature?

Partly

If applicable, is the statistical analysis and its interpretation appropriate?

No

Are all the source data underlying the results available to ensure full reproducibility?

Partly

Is the study design appropriate and is the work technically sound?

No

Are the conclusions drawn adequately supported by the results?

No

Are sufficient details of methods and analysis provided to allow replication by others?

No

Reviewer Expertise:

Water quality chemical assessment

I confirm that I have read this submission and believe that I have an appropriate level of expertise to confirm that it is of an acceptable scientific standard, however I have significant reservations, as outlined above.

# Response

**Published:** 2025-02-20

**Authors:** Kshyana Prava Samal

Dear Sir,

 Thank you, for your valuable feedback and suggestions to improve the manuscript.

 KIndly find the Answers.

1. The manuscript is short and may be short communication. Overall each section needs improvements.

The data collection from 67 wards in Bhubaneswar represents a comprehensive study, and all the data has been submitted to the f1000 research database repository on Figshare ( https://figshare.com/s/56b8ea8ad54c5449cc9d). Physico-chemical parameters have been tested at a minimum of five locations within each ward and are presented for analysis. Therefore, the data appears to be suitable for the study.

1. The real objectives may given for to address within the introduction.

 Already it is mentioned in the introduction as well as in Abstract.

1. Those tests conducted in present work, may only be basis of objectives.

Yes

1. Table 2 guideline of EC is not mg/l, check other units and limits.

Unit will be µs/Cm and have been changed in the manuscript.

1. The nitrate 30 mg/l is quite high in rivers, It may checked, the source of pollution in ice melt water.

The nitrate concentration of 30 mg/l could be attributed due to improper disposal of waste along the network.

1. The source of water at within rivers that did not compared before wards.

The comment raised by your kind good self is appreciated. The raw water get treated through a conventional water treatment plan prior distribution to the pipe line network. Therefore, the water quality of source is not compared with wards.

1. Inter river comparison may also present of three rivers.

Treated water is distributed in the pipeline network. The scope of the project was to analyse contamination within distribution system and not at the source. Therefore, inter river water quality comparison has not been conducted. This suggestion will be considered in our next phase of research. 

1. The methodology of parameters i.e nitrate may also explained. Therefore, WQI within good quality is suspected and may checked.

The test has been carried out referring standard method stated in APHA 2022.

1. The results of analysis may compared for statistical analysis, i.e t-test, PCA, Hierarchal analysis, piper diagram, SAR for irrigation.

The project was sponsored by Indian Council of Social Science Research (ICSSR) and the study area was limited to Bhubaneswar city for analysis of pipe water supply quality within the wards. Because of the time and space constraint we have limited our analysis with very few statistical parameter. As suggested by your good self-other statistical analysis will be considered in our next phase of study.

1. The table for physico chemical parameters may also given supplementary.

Already given in repository.( https://figshare.com/s/56b8ea8ad54c5449cc9d).

1. The discussion is very short and the number parameters were also less included, i.e metals.

The study was focused on physio chemical parameters analysis.  Due to the huge study area of 67 wards of Bhubaneswar the  testing parameters had to reduce  to complete the work in the project time frame. However, since the source of water is surface i.e rivers possibility of heavy metals is trivial. Therefore, heavy metal parameters test is not included under this scope of study.

1. The abstract and conclusion should also improved.

Improved, The revised manuscript is shared for your kind information.

1. The results were not compared with local studies, and international rivers or water supply.

Beyond the objective of the project. However, this will be considered in our next phase of the study.

1. Importance of water if any public water, dependency of type of people on supply water that did not given in detail.

Ans: Mentioned  in the introduction section of the revised  manuscript .

1. The nature of water in different wards may  compared with other smart cities.

Incorporated in the revised  manuscript as mentioned below.

 Groundwater is being extracted by households to meet the demand of drinking water. There is possibility of geogenic contamination of groundwater which may impact overall human health. The lack of proper regulation on groundwater extraction results in overexploitation in Indian cities (Suhag, 2016). As a result, utilities supplement surface water from distant sources, requiring significant investments in transmission infrastructure development (Larsen et al., 2016). The study conducted by World Resource Institute in the year 2020 classifies cities based on surface water availability index into six categories, (i) extremely low (-0.149 to -0.025); (ii) low (-0.024 to0.044); (iii) low to medium (0.045 –0.122); (iv) medium to high (0.123–0.198); high (0.199 – 0.275) and extremely high (0.276 – 0.389). The cities like Bhubaneswar, Kolkata, Indore, Kochi are having surface water availability index between 0.199 to 0.278.

1. Why samples crossed the limits, what was the reasons and how public awareness. Indicate the samples with GPS and contaminated samples.

Because of the age old pipe line and waste water contamination, some samples quality parameter have crossed the permissible limilt.

 This finding of the paper shall be used by Public Health Engineering Department, Government to create public awareness, develop policy and take necessary measures to maintain the standard water quality parameters as per the IS 10500:2012 code. After submission of the report to the Government, necessary action will be taken at department label.

1. Indicate the mass balance and error.

Beyond the scope of the objective.

1. The spatial variation may also explained.

Explained in section 2.3.2

 Spatial distribution maps were used to illustrate the variations in water quality parameters across Bhubaneswar city. These maps were generated using ArcGIS 10.2.1 software, which offers a range of spatial interpolation techniques. While no single method is universally accurate for all types of studies (Bronowicka-Mielniczuk et al., 2019), research has shown that Kriging methods, particularly the Ordinary Kriging (OK) method, tend to provide more precise prediction surfaces compared to other approaches (Coulibaly and Becker, 2007; Shamsudduha, 2007; Ohmer et al., 2017). However, the Inverse Distance Weighting (IDW) method is the most commonly used deterministic technique, often compared with Kriging methods (Li and Heap, 2011; Bronowicka-Mielniczuk et al., 2019). The IDW method is particularly suitable for analyzing data with low spatial autocorrelation (Jie et al., 2013). As a result, the spatial autocorrelation of the water quality values was taken into account when selecting the most appropriate method, which in this case was IDW.

 Bronowicka-Mielniczuk, U., Mielniczuk, J., Obro´slak, R., Przystupa, W., 2019. A comparison of some interpolation techniques for determining spatial distribution of nitrogen compounds in groundwater. Int. J. Environ. Res. 13 (4), 679–687.

 Coulibaly, M., Becker, S., 2007. Spatial interpolation of annual precipitation in South Africa-comparison and evaluation of methods. pp.494–502 Water Int. 32 (3).

 This will be incorporated in manuscript.

  

1. Figures may improved with ranges to better view.

- **Is the work clearly and accurately presented and does it cite the current literature?**

Partly

- **Is the study design appropriate and is the work technically sound?**

No

- **Are sufficient details of methods and analysis provided to allow replication by others?**

No

- **If applicable, is the statistical analysis and its interpretation appropriate?**

No

- **Are all the source data underlying the results available to ensure full reproducibility?**

Partly

- **Are the conclusions drawn adequately supported by the results?**

No

  

1. The manuscript is short and may be short communication. Overall each section needs improvements.

The data collection from 67 wards in Bhubaneswar represents a comprehensive study, and all the data has been submitted to the f1000 research database repository on Figshare ( https://figshare.com/s/56b8ea8ad54c5449cc9d). Physico-chemical parameters have been tested at a minimum of five locations within each ward and are presented for analysis. Therefore, the data appears to be suitable for the study.

1. The real objectives may given for to address within the introduction.

 Already it is mentioned in the introduction as well as in Abstract.

1. Those tests conducted in present work, may only be basis of objectives.

Yes

1. Table 2 guideline of EC is not mg/l, check other units and limits.

Unit will be µs/Cm and have been changed in the manuscript.

1. The nitrate 30 mg/l is quite high in rivers, It may checked, the source of pollution in ice melt water.

The nitrate concentration of 30 mg/l could be attributed due to improper disposal of waste along the network.

1. The source of water at within rivers that did not compared before wards.

The comment raised by your kind good self is appreciated. The raw water get treated through a conventional water treatment plan prior distribution to the pipe line network. Therefore, the water quality of source is not compared with wards.

1. Inter river comparison may also present of three rivers.

Treated water is distributed in the pipeline network. The scope of the project was to analyse contamination within distribution system and not at the source. Therefore, inter river water quality comparison has not been conducted. This suggestion will be considered in our next phase of research. 

1. The methodology of parameters i.e nitrate may also explained. Therefore, WQI within good quality is suspected and may checked.

The test has been carried out referring standard method stated in APHA 2022.

1. The results of analysis may compared for statistical analysis, i.e t-test, PCA, Hierarchal analysis, piper diagram, SAR for irrigation.

The project was sponsored by Indian Council of Social Science Research (ICSSR) and the study area was limited to Bhubaneswar city for analysis of pipe water supply quality within the wards. Because of the time and space constraint we have limited our analysis with very few statistical parameter. As suggested by your good self-other statistical analysis will be considered in our next phase of study.

1. The table for physico chemical parameters may also given supplementary.

Already given in repository.( https://figshare.com/s/56b8ea8ad54c5449cc9d).

1. The discussion is very short and the number parameters were also less included, i.e metals.

The study was focused on physio chemical parameters analysis.  Due to the huge study area of 67 wards of Bhubaneswar the  testing parameters had to reduce  to complete the work in the project time frame. However, since the source of water is surface i.e rivers possibility of heavy metals is trivial. Therefore, heavy metal parameters test is not included under this scope of study.

1. The abstract and conclusion should also improved.

Improved, The revised manuscript is shared for your kind information.

1. The results were not compared with local studies, and international rivers or water supply.

Beyond the objective of the project. However, this will be considered in our next phase of the study.

1. Importance of water if any public water, dependency of type of people on supply water that did not given in detail.

Ans: Mentioned  in the introduction section of the revised  manuscript .

1. The nature of water in different wards may  compared with other smart cities.

Incorporated in the revised  manuscript as mentioned below.

 Groundwater is being extracted by households to meet the demand of drinking water. There is possibility of geogenic contamination of groundwater which may impact overall human health. The lack of proper regulation on groundwater extraction results in overexploitation in Indian cities (Suhag, 2016). As a result, utilities supplement surface water from distant sources, requiring significant investments in transmission infrastructure development (Larsen et al., 2016). The study conducted by World Resource Institute in the year 2020 classifies cities based on surface water availability index into six categories, (i) extremely low (-0.149 to -0.025); (ii) low (-0.024 to0.044); (iii) low to medium (0.045 –0.122); (iv) medium to high (0.123–0.198); high (0.199 – 0.275) and extremely high (0.276 – 0.389). The cities like Bhubaneswar, Kolkata, Indore, Kochi are having surface water availability index between 0.199 to 0.278.

1. Why samples crossed the limits, what was the reasons and how public awareness. Indicate the samples with GPS and contaminated samples.

Because of the age old pipe line and waste water contamination, some samples quality parameter have crossed the permissible limilt.

 This finding of the paper shall be used by Public Health Engineering Department, Government to create public awareness, develop policy and take necessary measures to maintain the standard water quality parameters as per the IS 10500:2012 code. After submission of the report to the Government, necessary action will be taken at department label.

1. Indicate the mass balance and error.

Beyond the scope of the objective.

1. The spatial variation may also explained.

Explained in section 2.3.2

 Spatial distribution maps were used to illustrate the variations in water quality parameters across Bhubaneswar city. These maps were generated using ArcGIS 10.2.1 software, which offers a range of spatial interpolation techniques. While no single method is universally accurate for all types of studies (Bronowicka-Mielniczuk et al., 2019), research has shown that Kriging methods, particularly the Ordinary Kriging (OK) method, tend to provide more precise prediction surfaces compared to other approaches (Coulibaly and Becker, 2007; Shamsudduha, 2007; Ohmer et al., 2017). However, the Inverse Distance Weighting (IDW) method is the most commonly used deterministic technique, often compared with Kriging methods (Li and Heap, 2011; Bronowicka-Mielniczuk et al., 2019). The IDW method is particularly suitable for analyzing data with low spatial autocorrelation (Jie et al., 2013). As a result, the spatial autocorrelation of the water quality values was taken into account when selecting the most appropriate method, which in this case was IDW.

 Bronowicka-Mielniczuk, U., Mielniczuk, J., Obro´slak, R., Przystupa, W., 2019. A comparison of some interpolation techniques for determining spatial distribution of nitrogen compounds in groundwater. Int. J. Environ. Res. 13 (4), 679–687.

 Coulibaly, M., Becker, S., 2007. Spatial interpolation of annual precipitation in South Africa-comparison and evaluation of methods. pp.494–502 Water Int. 32 (3).

 This will be incorporated in manuscript.

  

1. Figures may improved with ranges to better view.

- **Is the work clearly and accurately presented and does it cite the current literature?**

Partly

- **Is the study design appropriate and is the work technically sound?**

No

- **Are sufficient details of methods and analysis provided to allow replication by others?**

No

- **If applicable, is the statistical analysis and its interpretation appropriate?**

No

- **Are all the source data underlying the results available to ensure full reproducibility?**

Partly

- **Are the conclusions drawn adequately supported by the results?**

No
